# Effectiveness and safety of COVID-19 vaccines on maternal and perinatal outcomes: a systematic review and meta-analysis

**DOI:** 10.1136/bmjgh-2023-014247

**Published:** 2024-04-04

**Authors:** Silvia Fernández-García, Laura del Campo-Albendea, Dharshini Sambamoorthi, Jameela Sheikh, Karen Lau, Nana Osei-Lah, Anoushka Ramkumar, Harshitha Naidu, Nicole Stoney, Paul Sundaram, Paulomi Sengupta, Samay Mehta, Shruti Attarde, Sophie Maddock, Millie Manning, Zainita Meherally, Kehkashan Ansari, Heidi Lawson, Magnus Yap, Tania Kew, Andriya Punnoose, Chloe Knight, Eyna Sadeqa, Jiya Cherian, Sangamithra Ravi, Wentin Chen, Kate Walker, Keelin O’Donoghue, Madelon van Wely, Elizabeth van Leeuwen, Elena Kostova, Heinke Kunst, Asma Khalil, Vanessa Brizuela, Edna Kara, Caron Rahn Kim, Anna Thorson, Olufemi T Oladapo, Lynne Mofenson, Sami L Gottlieb, Mercedes Bonet, Ngawai Moss, Javier Zamora, John Allotey, Shakila Thangaratinam

**Affiliations:** 1 WHO Collaborating Centre for Global Women’s Health, Institute of Metabolism and Systems Research, University of Birmingham, Birmingham, UK; 2 Clinical Biostatistics Unit, Hospital Universitario Ramón y Cajal, Madrid, Spain; 3 CIBERESP, Madrid, Spain; 4 University of Birmingham College of Medical and Dental Sciences, Birmingham, UK; 5 University of Nottingham, Nottingham, UK; 6 University of Aberdeen, Aberdeen, UK; 7 University College Cork, Cork, Ireland; 8 Amsterdam UMC Location AMC Center for Reproductive Medicine, Amsterdam, The Netherlands; 9 Amsterdam UMC Location AMC Department of Obstetrics Gynecology, Amsterdam, The Netherlands; 10 Queen Mary University of London Blizard Institute, London, UK; 11 Barts Health NHS Trust, London, UK; 12 St George’s University of London, London, UK; 13 Department of Reproductive Health and Research, World Health Organization, Geneve, Switzerland; 14 Research, Elizabeth Glaser Pediatric AIDS Foundation, Washington, District of Columbia, USA; 15 Katie's Team, London, UK; 16 NIHR Birmingham Biomedical Centre (BRC), University Hospitals Birmingham, Birmingham, UK; 17 Birmingham Women’s and Children’s NHS Foundation Trust, Birmingham, UK

**Keywords:** COVID-19, vaccines, obstetrics

## Abstract

**Objective:**

To assess the effects of COVID-19 vaccines in women before or during pregnancy on SARS-CoV-2 infection-related, pregnancy, offspring and reactogenicity outcomes.

**Design:**

Systematic review and meta-analysis.

**Data sources:**

Major databases between December 2019 and January 2023.

**Study selection:**

Nine pairs of reviewers contributed to study selection. We included test-negative designs, comparative cohorts and randomised trials on effects of COVID-19 vaccines on infection-related and pregnancy outcomes. Non-comparative cohort studies reporting reactogenicity outcomes were also included.

**Quality assessment, data extraction and analysis:**

Two reviewers independently assessed study quality and extracted data. We undertook random-effects meta-analysis and reported findings as HRs, risk ratios (RRs), ORs or rates with 95% CIs.

**Results:**

Sixty-seven studies (1 813 947 women) were included. Overall, in test-negative design studies, pregnant women fully vaccinated with any COVID-19 vaccine had 61% reduced odds of SARS-CoV-2 infection during pregnancy (OR 0.39, 95% CI 0.21 to 0.75; 4 studies, 23 927 women; I^2^=87.2%) and 94% reduced odds of hospital admission (OR 0.06, 95% CI 0.01 to 0.71; 2 studies, 868 women; I^2^=92%). In adjusted cohort studies, the risk of hypertensive disorders in pregnancy was reduced by 12% (RR 0.88, 95% CI 0.82 to 0.92; 2 studies; 115 085 women), while caesarean section was reduced by 9% (OR 0.91, 95% CI 0.85 to 0.98; 6 studies; 30 192 women). We observed an 8% reduction in the risk of neonatal intensive care unit admission (RR 0.92, 95% CI 0.87 to 0.97; 2 studies; 54 569 women) in babies born to vaccinated versus not vaccinated women. In general, vaccination during pregnancy was not associated with increased risk of adverse pregnancy or perinatal outcomes. Pain at the injection site was the most common side effect reported (77%, 95% CI 52% to 94%; 11 studies; 27 195 women).

**Conclusion:**

COVID-19 vaccines are effective in preventing SARS-CoV-2 infection and related complications in pregnant women.

**PROSPERO registration number:**

CRD42020178076.

**WHAT IS ALREADY KNOWN ON THIS TOPIC**

- Pregnant women with COVID-19 are at high risk of severe disease and death.
- Pregnant women were not included in vaccine trials, resulting in a lack of data on efficacy and safety leading to vaccine hesitancy.
- Existing reviews of observational studies do not account for confounding effects when combining studies, resulting in biased estimates and decreased confidence in findings.

**WHAT THIS STUDY ADDS**

- Analysis of adjusted data by confounding variables implies the control of sources of bias, such as the differences in healthcare-seeking behaviour.
- Fully vaccinated pregnant women are at reduced risk of having SARS-CoV-2 infection and being admitted to the hospital compared with unvaccinated pregnant women.
- Unvaccinated pregnant women are more likely to experience hypertensive disorders and caesarean sections, and their neonates are more likely to be admitted to a neonatal unit.

**HOW THIS STUDY MIGHT AFFECT RESEARCH, PRACTICE OR POLICY**

- Pregnant women should be counselled and reassured about the safety and benefits of COVID-19 vaccination during pregnancy, both for their own health and that of their babies.
- As the pace of the pandemic continues to evolve, the effectiveness of COVID-19 vaccines against new variants and the duration of protection they provide should be monitored.

## Introduction

Pregnant and recently pregnant women with SARS-CoV-2 infection are more likely to have severe COVID-19 disease and related mortality and morbidity than non-pregnant women of reproductive age.^1^ Globally, vaccination has been the most important intervention in preventing COVID-19-related mortality and morbidity in the general population.^2^ However, most phase III trials of COVID-19 vaccines excluded pregnant women, resulting in a lack of trial data on the safety and efficacy of these vaccines during pregnancy.^3^ Additionally, concerns about maternal and offspring outcomes have contributed to pregnant women’s reluctance to receive COVID-19 vaccination, despite current recommendations that pregnant women should receive the vaccine.^4 5^

Early observational studies on vaccine effectiveness focused on reporting the effects of any COVID-19 vaccine in pregnancy on maternal SARS-CoV-2 infection.^6–8^ Subsequent reviews reporting pregnancy outcomes varied in their inclusion of studies, overlapped their search periods by only a few months and were rapidly outdated, limiting their relevance.^9–12^ Some reviews only included studies from specific regions or countries and did not provide a global outlook.^13^ Existing reviews on the effects of vaccines on pregnant women only included aggregate data and did not adjust for confounding variables, which implied they were not controlled for some sources of bias such as the differences in healthcare-seeking behaviour.^9 13^

We undertook a systematic review to comprehensively assess the effects of any COVID-19 vaccines administered to pregnant women before or during pregnancy on infection-related, pregnancy-related maternal and offspring and reactogenicity outcomes.

## Methods

Our prospectively registered protocol (PROSPERO CRD42020178076) on effects of SARS-CoV-2 in pregnancy was extended to evaluate the effects of COVID-19 vaccines on infection-related and pregnancy-related maternal and offspring outcomes.^14^ We report our review using the Preferred Reporting Items for Systematic Reviews and Meta-Analyses guidance (see online supplemental appendix 1).

10.1136/bmjgh-2023-014247.supp1 Supplementary data

### Literature search

We searched major databases, preprint servers and websites that serve as repositories for COVID-19 studies, including Medline, Embase, Cochrane database, WHO COVID-19 database, Living Overview of the Evidence platform, China National Knowledge Infrastructure and Wanfang databases for relevant studies on COVID-19 in pregnant women (1 December 2019 to 30 January 2023). We coordinated our search efforts with the WHO Library, and the Cochrane Gynaecology and Fertility group. We contacted established groups coordinating or conducting surveillance and studies in pregnant women receiving COVID-19 vaccination, such as the US Centers for Disease Control and Prevention and the European Centre for Disease Prevention and Control, for information on published and upcoming data. Additional searches of preprint servers, blogs, websites that serve as repositories, social media, guidelines and reference lists of included studies were conducted.^15^ No language restrictions were applied. Online supplemental appendix 2 provides details of the search strategies and databases.

10.1136/bmjgh-2023-014247.supp2 Supplementary data

### Study selection

Nine pairs of independent reviewers selected studies using a two-stage process. The reviewers first screened the titles and abstracts of studies and then assessed the full text of the selected studies in detail for eligibility. Disagreements between reviewers were resolved through discussion with a third reviewer (ST, JA or SF-G). We included test-negative design studies, and comparative cohorts reporting adjusted and unadjusted effects of any COVID-19 vaccine received by women before or during pregnancy on infection-related, pregnancy-related maternal and offspring outcomes, and the rates of reactogenicity outcomes. In test-negative design studies, the source population was pregnant women with COVID-19-like illness, and outcomes of interest were maternal SARS-CoV-2 infection, severe disease and maternal hospital admission outcomes. In neonates with COVID-19-like illness, our outcome was neonatal SARS-CoV-2 infection. SARS-CoV-2 infection was diagnosed by laboratory testing. Those who tested positive were considered as cases, and those who tested negative were controls, and their vaccination status assessed. For infection-related outcomes, we only included studies where women received a complete schedule of the COVID-19 vaccine during pregnancy; for pregnancy-related maternal and offspring outcomes, women were included if they received at least one dose during pregnancy, except for miscarriage outcome where women vaccinated before pregnancy were included. We additionally included non-comparative cohort and case-control studies with a minimum of 10 participants if they reported on reactogenicity outcomes of COVID-19 vaccines in women vaccinated during pregnancy. We excluded case reports and case series, and studies where women were vaccinated after pregnancy.

### Study quality assessment and data extraction

Two independent reviewers (SF-G, LdC-A) assessed the quality of the comparative cohort studies and test-negative design case-control studies in our primary analysis using the ‘Risk of Bias in Non-Randomised Studies of Interventions’ (ROBINS-I) tool.^16^ We used a prepiloted form to extract information on study design, recruitment period, predominant circulating SARS-CoV-2 variant at the time of study, setting (hospital, country), World Bank region, details of key adjustment variables (age, body mass index (BMI), gestational age, education, diabetes, chronic hypertension), the vaccine platform and vaccine product administered, the number of doses and time of vaccination (before or during pregnancy and trimester). The number of doses was assumed to be ‘at least one dose’ when the number received was unclear or when women included had received different doses. We considered the group to be ‘partially vaccinated’ when women received only one dose of two-dose vaccines and ‘fully vaccinated’ when they received one dose of single-dose vaccines or two doses of vaccines requiring two doses for immunogenicity. When women received three doses, we considered the group as ‘booster dose’.

We extracted data on the adjusted estimate of the effect of COVID-19 vaccines, the number of vaccinated and non-vaccinated pregnant women and the number of events for infection-related maternal outcomes such as diagnosis of maternal SARS-CoV-2 infection before delivery, maternal hospital admission, maternal death and maternal severe COVID-19 disease defined as admission to the intensive care unit (ICU), hospitalisation due to severe disease or as defined by study authors; infection-related offspring outcomes like offspring SARS-CoV-2 infection up to 6 months after delivery; pregnancy-related maternal outcomes included miscarriage, preterm birth <37 weeks, caesarean section, postpartum haemorrhage, gestational diabetes and hypertensive disorders and offspring outcomes included stillbirth, neonatal death, neonatal intensive care unit (NICU) admission, low 5 min Apgar score (<7) and small-for-gestational-age baby. We extracted data on the number of vaccinated pregnant women who reported reactogenicity outcomes such as headache, fever, myalgia, fatigue and pain at injection site from comparative and non-comparative cohorts and case-control studies. We did not consider the booster doses for reactogenicity outcomes.

### Statistical analysis

Our primary analysis was based on test-negative design and comparative cohort studies with adjusted analyses reporting the effects of COVID-19 vaccines on infection-related, and pregnancy-related maternal and offspring outcomes. We pooled the adjusted estimates using random effects meta-analysis and summarised the findings as HRs, risk ratios (RRs) or ORs with 95% CIs.

For the secondary analysis, we pooled data from all included comparative cohort studies with unadjusted estimates and summarised the findings of infection-related and pregnancy-related maternal and offspring outcomes as ORs with 95% CIs. We calculated the rates of reactogenicity outcomes from non-comparative studies as proportions with 95% CIs using DerSimonian and Laird random-effects meta-analysis, after transforming data using Freeman-Tukey double-arcsine transformation. Heterogeneity was reported using I^2^. All statistical analyses were performed using Stata (V.18).

### Patient and public involvement

This study is supported by Katie’s team, a dedicated patient and public involvement group in women’s health. The team was involved in the interpretation and reporting of this systematic review through participation in virtual meetings. Findings will be made available on our website in a format more suitable for patients and members of the public (www.birmingham.ac.uk/research/who-collaborating-centre/pregcov/index.aspx).

## Results

**Figure 1:**
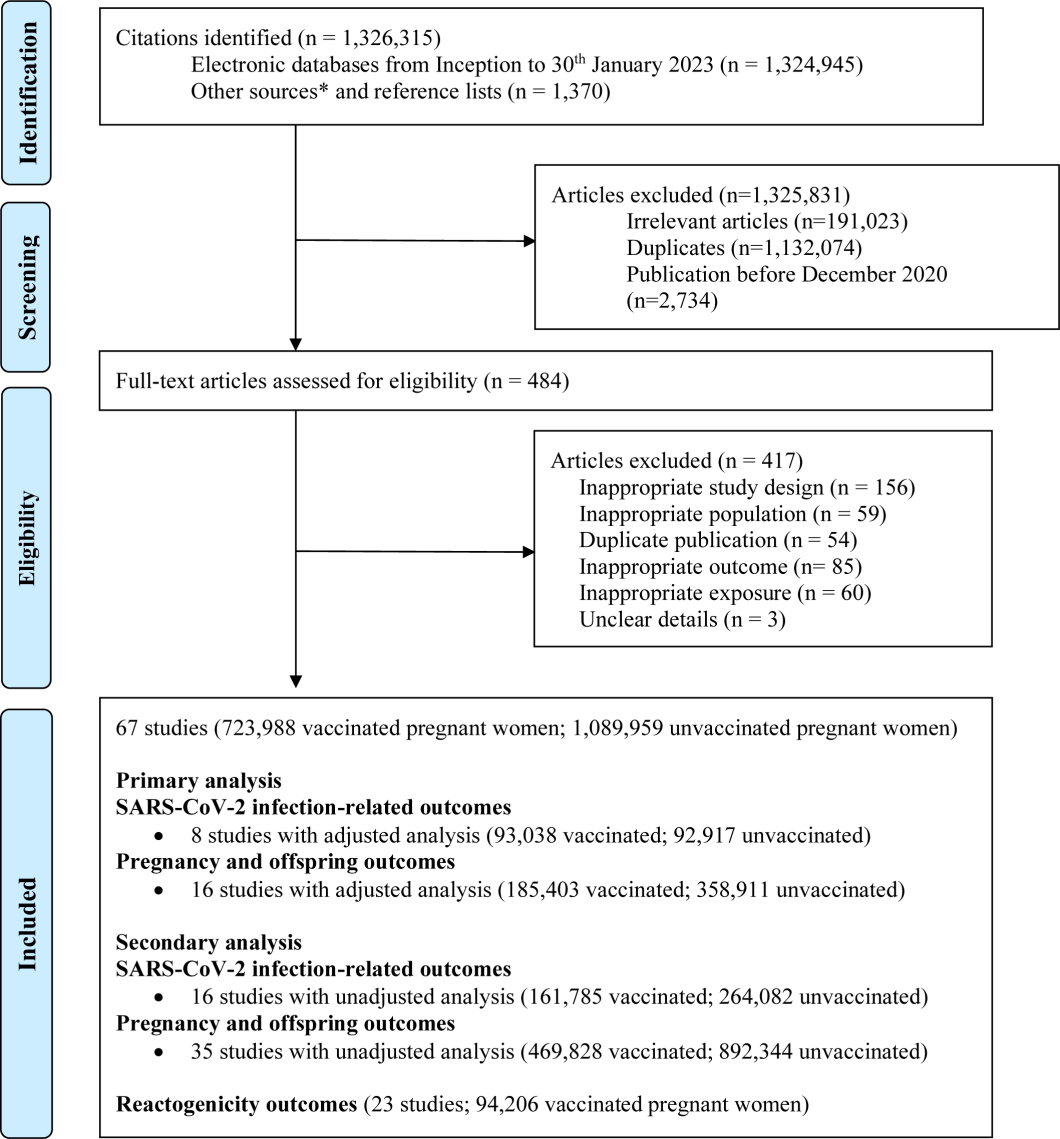
Study selection process in the systematic review. Created and owned by the authors. *Twitter, national reports, blog Thornton J, ObG Project, COVID-19 and Pregnancy Cases (https://www.obgproject.com/2020/04/07/covid-19-research-watch-with-dr-jim-thornton/); EPPI-Centre, COVID-19: a living systematic map of evidence (http://eppi.ioe.ac.uk/cms/Projects/DepartmentofHealthandSocialCare/Publishedreviews/COVID19Livingsystematicmapoftheevidence/tabid/3765/Default.aspx); Norwegian Institute of Public Health (NIPH), NIPH systematic and living map on COVID-19 evidence (https://www.nornesk.no/forskningskart/NIPH_mainMap.html); John Hopkins University Center for Humanitarian Health; COVID-19, Maternal and Child Health, Nutrition (http://hopkinshumanitarianhealth.org/empower/advocacy/covid-19/covid-19-children-and-nutrition/); ResearchGate, COVID-19 research community (https://www.researchgate.net/community/COVID-19); Living Overview of the Evidence, COVID-19 (https://app.iloveevidence.com/loves/5e6fdb9669c00e4ac072701d?population=5d062d5fc80dd41e58ba8459).

We included 67 studies (1 813 947 women) from 1 326 315 identified articles (figure 1). Twenty-four were included in the primary analysis, with eight performing adjusted analysis (185 955 women) for SARS-CoV-2 infection-related outcomes.^6–8 17–21^ Six of them reported maternal SARS-CoV-2 infection, three reported maternal hospital admission and two reported severe COVID-19 disease and neonatal SARS-CoV-2 infection. Sixteen performed adjusted analysis for pregnancy-related maternal and offspring outcomes (544 314 women).^22–37^ We included 16 studies (425 867 women) reporting SARS-CoV-2 infection-related outcomes^6 7 17 19 21 31 33 36 38–45^ and 35 (1 362 172 women) reporting pregnancy-related maternal and offspring outcomes in the secondary analysis.^17 18 21–24 29–34 36 38 39 41–60^ Twenty-three studies reported reactogenicity outcomes (94 206 women) following vaccination.^38 39 46 61–80^

### Characteristics of the included studies

A third of the included studies were from the Middle East and North Africa (22/67; 193 889 women), followed by North America (28%, 19/67; 397 756 women), Europe and Central Asia (22.5%, 15/67; 1 150 470 women), East Asia and Pacific (10.5%, 7/67; 42 204 women) and Latin America and Caribbean (3%, 2/67; 22 122 women), South Asia (1.5%, 1/67; 247 women) and one was a multicountry study (1.5%, 1/67; 4618 women). Fifty-nine studies were from high-income countries (59/67; 1 782 548 women), six from upper-middle-income countries (6/67; 26 534 women), one from lower-middle-income countries (1/67; 247 women) and one from a mix of high-income, upper-middle-income and lower-middle-income countries (1/67; 4618). Overall, 45 studies included women vaccinated with mRNA vaccine only (281 030 women), four studies included inactivated virus (3088 women), one study viral vector vaccine (247 women), 14 studies mRNA and/or viral vector vaccines (436 453 women), one mRNA, viral vector and inactivated virus vaccines (2886 women) and two did not report the type of vaccine (284 women). Most of the studies included in the primary analysis were adjusted by maternal age (88%, 21/24), followed by diabetes (42%, 10/24), hypertension (33%, 8/24), BMI (33%, 8/24), gestational age (17%, 4/24) and education (4%, 1/24). Three of the eight studies performing adjusted analysis for SARS-CoV-2 infection-related outcomes were from the Delta and Omicron periods (134 779 women), one study was from the Delta period (464 women), one from the Omicron period (4618 women), one from the Alpha and Beta periods (4534 women), one from the Alpha period and other variants (21 722 women) and one from the Delta period and other variants (19 838 women). Online supplemental appendix 3 describes the characteristics of all included studies.

10.1136/bmjgh-2023-014247.supp3 Supplementary data

### Quality of studies included in primary analysis

**Figure 2:**
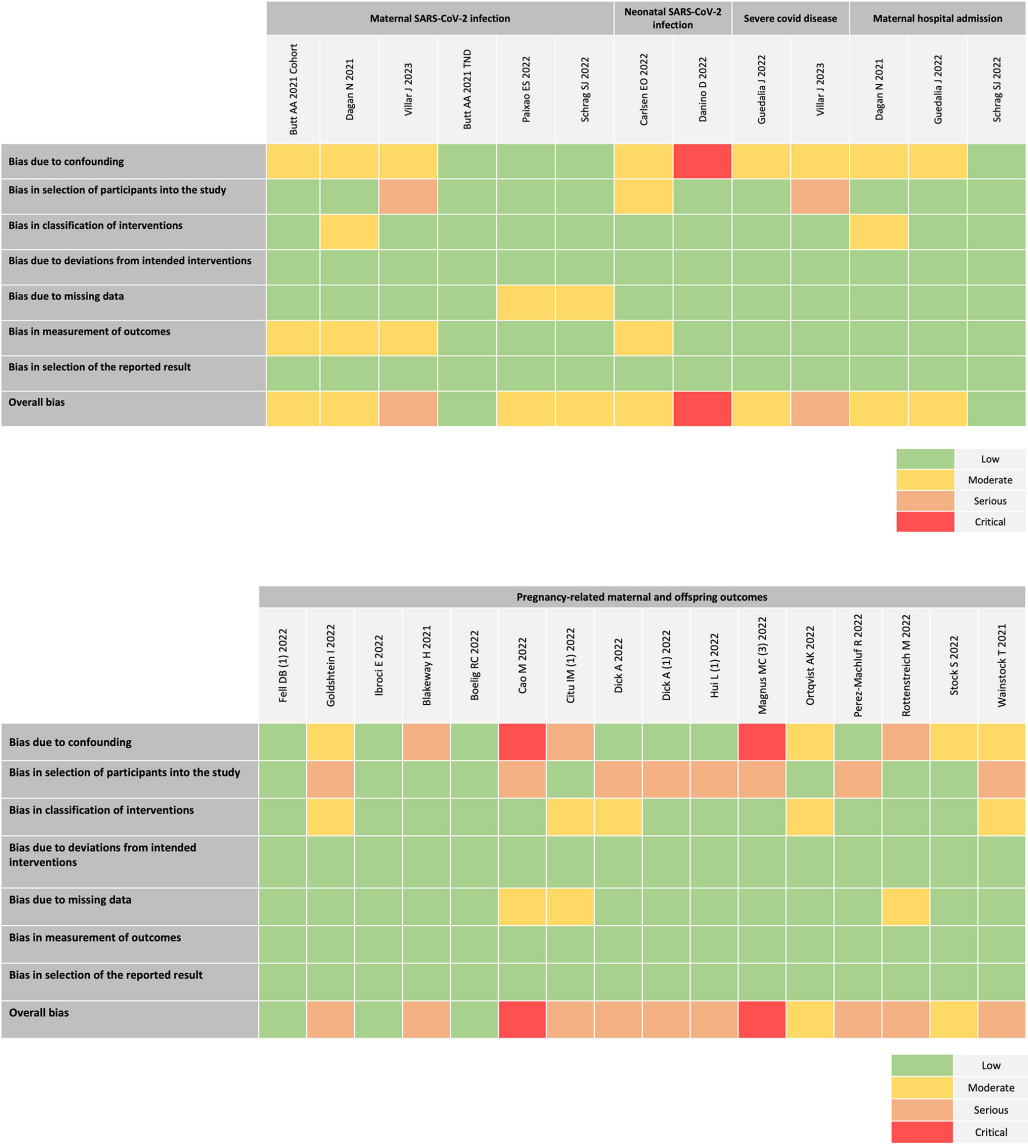
Quality assessment for risk of bias in studies of primary analysis using Risk of Bias in Non-Randomised Studies of Interventions tool. Created and owned by the authors.

Figure 2 provides the risk of bias for the included test-negative design and adjusted cohort studies included in the main analysis. For the maternal SARS-CoV-2 infection outcome, 17% of studies (1/6) were considered to be low risk, 66% (4/6) moderate risk and 17% (1/6) as serious risk. Of the two studies reporting severe COVID-19 disease, one was considered to be moderate risk and the other serious. For maternal hospital admission outcome, two studies were classified as having moderate risk and one as low risk. Of the two studies reporting neonatal SARS-CoV-2 infection, one study was considered to have critical risk of bias rating, as prematurity, a postintervention variable was used as an adjustment factor.^18^ More than half of the studies reporting pregnancy-related maternal and offspring outcomes were considered to be serious risk (9/16), 19% (3/16) low risk and 12% (2/16) as moderate or critical risk. Online supplemental appendix 4 describes the consensus judgements used to assign the risk of bias in each domain.

10.1136/bmjgh-2023-014247.supp4 Supplementary data

### Effects of COVID-19 vaccines on SARS-CoV-2 infection-related outcomes

**Figure 3:**
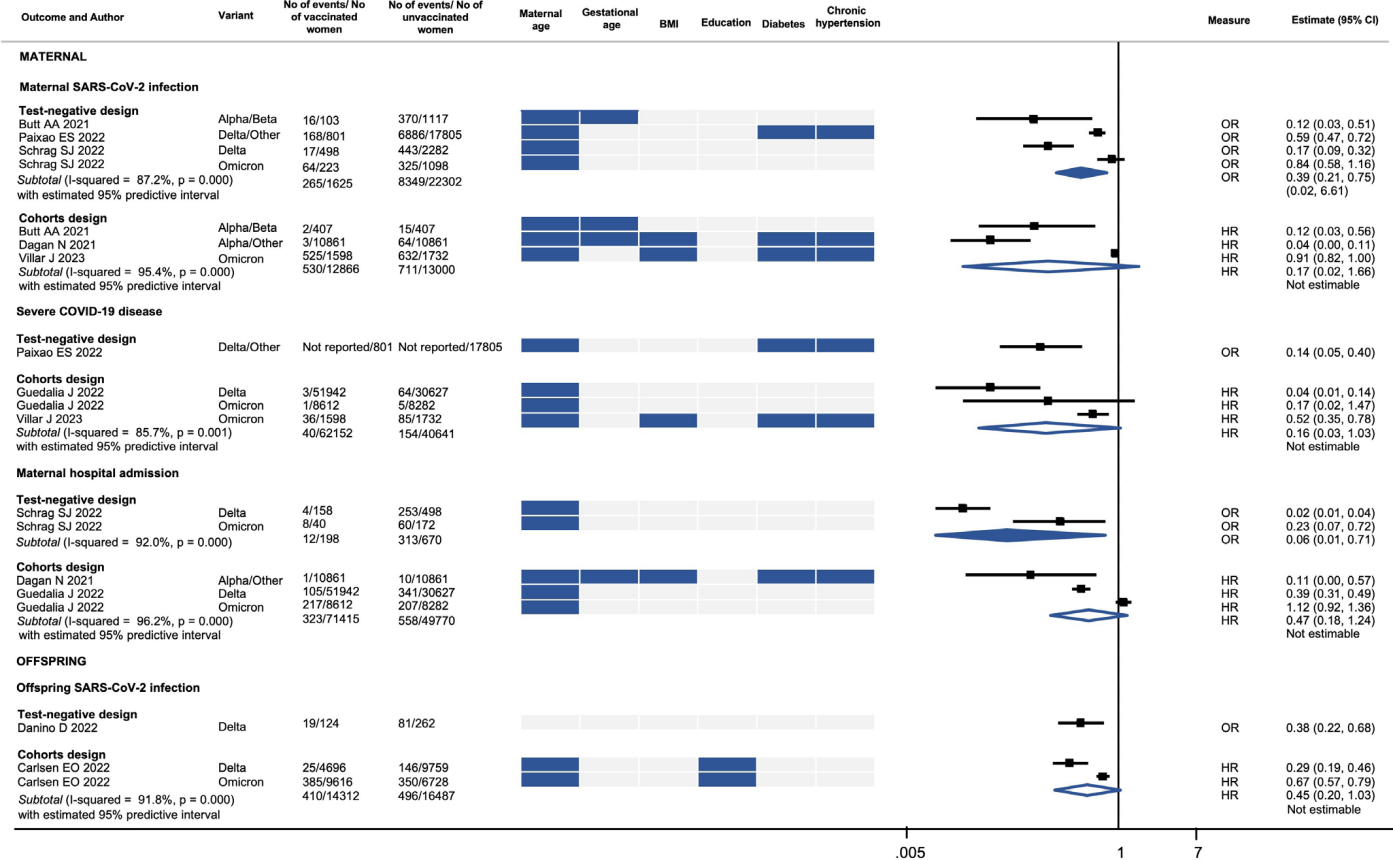
Vaccine effectiveness for SARS-CoV-2 infection-related outcomes. BMI, body mass index. Created and owned by the authors.

In our primary analysis of test-negative design studies, women who were fully vaccinated had a 61% reduction in the odds of SARS-CoV-2 infection during pregnancy (OR 0.39, 95% CI 0.21 to 0.75; 4 studies, 23 927 women; I^2^=87.2%) and a 94% reduction in the odds of hospital admission (OR 0.06, 95% CI 0.01 to 0.71; 2 studies, 868 women; I^2^=92%) (figure 3). The effect of the vaccines on infection-related outcomes of the adjusted comparative cohort studies is imprecise and heterogeneous. Although it consistently shows a reduction in the hazard of infection-related outcomes, this reduction does not reach statistical significance (figure 3). We did not find any test-negative design study or adjusted comparative cohort study reporting on maternal death. Table 1 provides the summary estimates of the effects of COVID-19 vaccines reported in test-negative design studies (adjusted), comparative cohort (adjusted) and unadjusted cohort studies. Online supplemental appendix 5 provides details of individual unadjusted cohort studies.

10.1136/bmjgh-2023-014247.supp5 Supplementary data

**Table 1:** Summary estimates reported in test-negative design (adjusted), comparative cohort (adjusted) and comparative cohort (unadjusted) studies

Outcome	Test-negative design (adjusted)			Comparative cohort (adjusted)			Comparative cohort (unadjusted)		
	No. of studies (women)	HR (95% CI)	I^2^ (%)	No. of studies (women)	Estimate (95% CI)	I^2^ (%)	No. of studies (women)	OR (95% CI)	I^2^ (%)
SARS-CoV-2 infection-related outcomes									
Maternal SARS-CoV-2 infection	4 (23 927)	0.39 (0.21 to 0.75)	87.2	3 (25 866)	OR 0.17 (0.02 to 1.66)	95.4	11 (397 679)	0.63 (0.47 to 0.85)	98.5
Severe COVID-19 disease	1 (18 606)	0.14 (0.05 to 0.40)		3 (102 793)	OR 0.16 (0.03 to 1.03)	85.7	11 (132 759)	0.47 (0.22 to 0.97)	80.9
Maternal hospital admission	2 (868)	0.06 (0.01 to 0.71)	92	3 (121 185)	OR 0.47 (0.18 to 1.24)	96.2	2 (36 782)	0.41 (0.13 to 1.28)	92
Offspring SARS-CoV-2 infection	1 (386)	0.38 (0.22 to 0.68)		2 (30 799)	OR 0.45 (0.20 to 1.03)	91.8	3 (31 848)	0.52 (0.33 to 0.82)	87.6
Maternal death							9 (148 297)	0.53 (0.12 to 2.47)	64.4
Pregnancy-related maternal outcomes									
Miscarriage				4 (43 465)	OR 0.96 (0.90 to 1.04)	0	3 (1113)	1.60 (0.70 to 1.91)	0
Preterm birth <37 weeks				5 (25 516)	OR 0.79 (0.59 to 1.06)	68.3	21 (1 104 043)	0.90 (0.83 to 0.97)	75
				1 (24 190)	RR 0.95 (0.83 to 1.10)				
Caesarean section				6 (30 192)	OR 0.91 (0.85 to 0.98)	0	15 (188 144)	1.11 (1.03 to 1.20)	48.6
				2 (54 569)	RR 0.94 (0.81 to 1.08)	34.9			
Postpartum haemorrhage				5 (30 192)	OR 1.49 (0.91 to 2.44)	86.7	6 (104 693)	0.82 (0.68 to 1.00)	0
				1 (52 775)	RR: 0.90 (0.81 to 1.00)				
Gestational diabetes				1 (5618)	OR 1.10 (0.90 to 1.30)		11 (263 319)	1.04 (0.89 to 1.21)	94.2
				2 (115 085)	RR 1.17 (1.14 to 1.20)	0			
Hypertensive disorders				5 (15 739)	OR 1.11 (0.87 to 1.43)	0	10 (217 486)	1.13 (1.02 to 1.25)	49
				2 (115 085)	RR 0.88 (0.85 to 0.92)	0			
Pregnancy-related offspring outcomes									
Stillbirth				2 (17 907)	OR 0.38 (0.09 to 1.59)	89.4	11 (1 024 952)	0.78 (0.65 to 0.92)	36.5
Admission to neonatal intensive care unit				4 (173 978)	OR 0.88 (0.71 to 1.08)	37.9	9 (108 534)	0.82 (0.79 to 0.86)	0
				2 (54 569)	RR 0.92 (0.87 to 0.97)	0			
Low 5 min Apgar score <7				4 (179 034)	OR 0.89 (0.73 to 1.08)	29.3	9 (113 540)	0.89 (0.81 to 0.99)	0
				1 (51 922)	RR 0.88 (0.77 to 1.01)				
Small for gestational age				6 (172 483)	OR 0.96 (0.90 to 1.02)	0	8 (153 813)	0.99 (0.95 to 1.03)	0
				1 (24 190)	RR 0.97 (0.87 to 1.08)				
Neonatal death				1 (24 190)	RR 0.84 (0.43 to 1.72)				

Created and owned by the authors.

*As reported in the individual studies, adjusted cohort results for pregnancy-related maternal and offspring outcomes are shown as OR or RR.

RR, risk ratio.

### Effects of COVID-19 vaccines on pregnancy-related maternal and offspring outcomes

Meta-analysis of adjusted comparative cohort studies showed a 12% reduction in the risk of hypertensive disorders in pregnancy (RR 0.88, 95% CI 0.82 to 0.92; 2 studies; 115 085 women) in women vaccinated versus not vaccinated in pregnancy. The odds of caesarean section (OR 0.91, 95% CI 0.85 to 0.98; 6 studies; 30 192 women) was reduced in the pooled analysis of adjusted comparative cohorts. We did not find any association between COVID-19 vaccination and other maternal outcomes, except for gestational diabetes (table 1). We observed an 8% reduction in the risk of newborn’s admission to the NICU (RR 0.92, 95% CI 0.87 to 0.97; 2 studies; 54 569 women) in babies born to vaccinated versus not vaccinated women. There were no significant differences observed in other offspring outcomes (table 1). The summary findings of data from adjusted and unadjusted cohort studies for pregnancy-related maternal and offspring outcomes are provided in online supplemental appendices 6 and 7. The summary findings from the adjusted individual studies are provided in online supplemental appendices 8 and 9.

10.1136/bmjgh-2023-014247.supp6 Supplementary data

10.1136/bmjgh-2023-014247.supp7 Supplementary data

10.1136/bmjgh-2023-014247.supp8 Supplementary data

10.1136/bmjgh-2023-014247.supp9 Supplementary data

### Vaccination in pregnancy and reactogenicity outcomes

The most common side effects reported by pregnant women vaccinated with any number of doses of COVID-19 vaccine were mild pain at the injection site (77%, 95% CI 52% to 94%; 11 studies; 27 195 women), followed by fatigue (29%, 95% CI 15% to 46%; 14 studies; 72 671 women) (table 2). Other side effects, such as headache and myalgia, were reported by 12% of vaccinated pregnant women each, while fever was reported by 5% (95% CI 2% to 8%; 19 studies; 82 972 women) of vaccinated pregnant women (table 2).

**Table 2:** Reactogenicity outcomes in pregnant women vaccinated for COVID-19

Side effects	Partially vaccinated				Fully vaccinated				Any number of doses			
	No. of studies	No. of events	Proportion (95% CI)	I^2^ (%)	No. of studies	No. of events	Proportion (95% CI)	I^2^ (%)	No. of studies	No. of events	Proportion (95% CI)	I^2^ (%)
Fever	13	1683/36 439	0.06 (0.03 to 0.10)	99.2	14	8158/28 139	0.16 (0.07 to 0.26)	99.7	19	1766/82 972	0.05 (0.02 to 0.08)	99.5
Headache	10	3987/28 491	0.10 (0.05 to 0.17)	99.4	13	9207/21 999	0.20 (0.09 to 0.34)	99.7	17	4885/40 751	0.12 (0.06 to 0.18)	99.7
Myalgia	8	2208/23 392	0.09 (0.04 to 0.15)	98.8	11	7376/17 345	0.28 (0.12 to 0.47)	99.7	13	2789/27 920	0.12 (0.08 to 0.17)	98.6
Fatigue	8	6727/22 827	0.26 (0.23 to 0.29)	86.9	10	12 751/18 746	0.52 (0.45 to 0.60)	98.1	14	8042/72 671	0.29 (0.15 to 0.46)	99.9
Pain at injection site	7	20540/22 922	0.85 (0.76 to 0.93)	99.3	8	16 896/18 608	0.80 (0.73 to 0.85)	98.1	11	21 623/27 195	0.77 (0.52 to 0.94)	99.9

Created and owned by the authors.

## Discussion

COVID-19 vaccination in pregnant women reduces the risks of maternal SARS-CoV-2 infection and admission to the hospital during pregnancy. Vaccination in pregnancy appears to reduce risks of maternal hypertensive disorders during pregnancy, caesarean section and neonatal admission to ICU. Pain at injection site was the most common side effect of COVID-19 vaccination.

Our comprehensive review on the effects of COVID-19 vaccination in pregnant women provides robust data by focusing on test-negative design studies, which are a rigorous method to reduce the bias, and adjusted comparative cohorts in our main analysis. We used ROBINS-I tool that provides a comprehensive assessment of the risk of bias. We undertook an extensive deduplication process and minimised the risk of including duplicate data. By focusing on both SARS-CoV-2 infection-related and pregnancy-related maternal and offspring outcomes, we addressed questions that are important to women in making decisions regarding vaccination. The large sample size in our review allowed us to assess the magnitude of benefit and risk of harm with high precision, including for less common but important outcomes such as neonatal admission to ICU. We included studies from different regions and income levels, with no language restrictions.

Our review has some limitations. The trimester of exposure to vaccines was poorly reported in primary studies, which did not allow us to see the effect of the timing of vaccination on infection-related, pregnancy-related maternal and offspring or reactogenicity outcomes. We did not find any test-negative design or adjusted comparative cohort study reporting on maternal death. Some of the studies included women vaccinated before or during pregnancy and we were unable to separately give estimates for women vaccinated during pregnancy. We did not evaluate long-term effects of the vaccines and were unable to analyse data on adverse effects such as thrombocytopenia, embolic reactions or myocarditis due to the lack of enough studies reporting these outcomes. Similarly, the sample sizes and event numbers were small for outcomes such as miscarriage and maternal death requiring cautious interpretation. We found an association between vaccination and an increased risk of gestational diabetes, but this is based on two different populations from the same adjusted comparative cohort study.^35^ Further data are needed to confirm this. We were unable to assess the effects of vaccines on the different variants due to the few published papers reporting separately for periods of variants of concern. Despite our comprehensive search, most of the studies that met our inclusion criteria are from high-income countries and external validity of our findings may not be accurate for middle-income and low-income settings.

In pregnant women from test-negative design studies, we found a reduction in the odds of SARS-CoV-2 infection and hospital admission after complete vaccination. The findings are similar to those observed in clinical trials and real-world data showing COVID-19 vaccines to be effective in preventing SARS-CoV-2 infections, severe COVID-19 disease and deaths, in the general adult population.^81 82^ In general population, the effectiveness of COVID-19 vaccines varied depending on the type of vaccine, the population being vaccinated, the number of doses, the variant and the immunity of individuals.^82^ However, we refrained from performing this analysis as data were only limited to non-adjusted cohort studies, with high degree of bias. Previous reviews on COVID-19 vaccines in pregnancy often limited their reporting to a few specific regions or countries, or only on SARS-CoV-2 infection.^9 13^ In addition, most of these reviews did not include test-negative design studies or did not use data from adjusted comparative cohort studies analysis. Our findings, based on these study designs, are inherently controlled for some sources of bias, such as differences in healthcare-seeking behaviour and access by vaccination status and are less affected by confounding factors.^83^

COVID-19 vaccines are recommended for use in pregnancy by WHO, policymakers and professional bodies globally.^5 84–87^ The exclusion of pregnant women from the initial clinical trials limited the acquisition of safety data and the ability to make evidence-based recommendations at the early stages of vaccine implementation. Our study demonstrated that reactogenicity-related side effects of COVID-19 vaccine in pregnant women were generally mild, similar to those reported in the general population. Rare adverse events such as vaccine-associated thrombotic thrombocytopenia (incidence 0.73 cases per 100 000 vaccinated persons receiving adenovirus-based vaccines), myocarditis (12.6 cases per million doses messenger RNA (mRNA) vaccine) and Guillain-Barré syndrome (7.8 cases per million doses adenovirus vaccine) may not be captured, and a very large sample size would be needed to evaluate such rare events during pregnancy.^88^

Pregnant women should be counselled and reassured about the safety and benefits of COVID-19 vaccination during pregnancy, both for their own health and that of their babies. Our findings demonstrate the effectiveness and safety of different COVID-19 vaccines. Although most available data are for the mRNA vaccines Pfizer-BioNTech BNT162b2 and Moderna mRNA-1273, our review also includes data on Sinovac-CoronaVac, Sinopharm BIBP, Janssen Ad26.COV2.S, AZD ChAdOx1-S, Cansino Ad5-nCoV-S and Bharat BBV152 Covaxin. More data on these non-mRNA vaccines would strengthen existing findings. Women should discuss their individual risks and concerns with their healthcare provider, who can help reassure and support them in making the best decision about vaccination.

The response was too slow during the pandemic, and equitable and timely distribution of COVID-19 vaccines to all communities, particularly vulnerable populations, could have saved more lives at the height of the pandemic. Barriers to vaccine access, including transportation, language and technology barriers, should be addressed and ensure that vaccine distribution sites are located in areas that are easily accessible to underserved communities.^89^ An investment in providing vaccine education and outreach campaigns to promote acceptance and address hesitancy is critical. Close collaboration is needed between professional colleges and community organisations to provide accurate and appropriate information about vaccine safety and efficacy and continuous monitoring to provide updates to help build trust and confidence.

The virus has shown its ability to mutate, leading to the emergence of new variants. The effectiveness of existing vaccines against these variants is continuously monitored by vaccine manufacturers and health authorities. This has led to the recommendation of supplementary doses to enhance immunity or a single dose in each pregnancy, regardless of previous vaccination status.^90^ It is important to continue research on the effectiveness of COVID-19 vaccines against different variants of the virus, the duration of protection they provide and further safety data from non-mRNA vaccines. The Human Reproduction Programme (the United Nations Development Programme/United Nations Population Fund/UNICEF/WHO/World Bank Special Programme of Research, Development and Research Training in Human Reproduction) initiatives can be adapted and generalised to prepare for quicker response in future epidemics.^91^ The development of research infrastructure, which includes strengthening laboratories, research facilities and data management systems can be repurposed for epidemic outbreaks. In addition, collaboration with various stakeholders such as governments, non-governmental organisations and research institutions can facilitate faster response times and resource mobilisation. Research should also focus on identifying reasons for vaccine hesitancy, particularly among pregnant women.^92^ Effective communication strategies need to be developed to address these concerns.

## Conclusion

COVID-19 vaccination in pregnant women is highly effective in reducing the odds of maternal SARS-CoV-2 infection, and hospital admission, and improves pregnancy outcomes, with no serious safety concerns. The interpretation of our findings may be impacted by changes in vaccine recommendations and the changing landscape of SARS-CoV-2 variants.

### Dissemination to participants and related patient and public communities

The PregCOV-19 Living Systematic Review Group will disseminate the findings through a dedicated website (www.birmingham.ac.uk/research/who-collaborating-centre/pregcov/index.aspx) and social media.

## Data Availability

No data are available.

## References

[1] Allotey J , Stallings E , Bonet M , et al . Clinical manifestations, risk factors, and maternal and perinatal outcomes of Coronavirus disease 2019 in pregnancy: living systematic review and meta-analysis. BMJ 2020;370:m3320. 10.1136/bmj.m3320 32873575 PMC7459193

[2] Watson OJ , Barnsley G , Toor J , et al . Global impact of the first year of COVID-19 vaccination: a mathematical modelling study. Lancet Infect Dis 2022;22:1293–302. 10.1016/S1473-3099(22)00320-6 35753318 PMC9225255

[3] World Health Organization . COVID-19 vaccines technical documents. n.d. Available: https://www.who.int/groups/strategic-advisory-group-of-experts-on-immunization/covid-19-materials

[4] Bhattacharya O , Siddiquea BN , Shetty A , et al . COVID-19 vaccine hesitancy among pregnant women: a systematic review and meta-analysis. BMJ Open 2022;12:e061477. 10.1136/bmjopen-2022-061477 PMC9393853 35981769

[5] World Health Organization . SAGE updates COVID-19 vaccination guidance. 2023. Available: https://www.who.int/news/item/28-03-2023-sage-updates-covid-19-vaccination-guidance

[6] Butt AA , Chemaitelly H , Al Khal A , et al . SARS-CoV-2 vaccine effectiveness in preventing confirmed infection in pregnant women. J Clin Invest 2021;131:e153662. 10.1172/JCI153662 34618693 PMC8631593

[7] Dagan N , Barda N , Biron-Shental T , et al . Effectiveness of the BNT162b2 mRNA COVID-19 vaccine in pregnancy. Nat Med 2021;27:1693–5. 10.1038/s41591-021-01490-8 34493859

[8] Paixao ES , Wong KLM , Alves FJO , et al . Coronavac vaccine is effective in preventing symptomatic and severe COVID-19 in pregnant women in Brazil: a test-negative case-control study. BMC Med 2022;20:146. 10.1186/s12916-022-02353-w 35379250 PMC8979723

[9] Ma Y , Deng J , Liu Q , et al . Effectiveness and safety of COVID-19 vaccine among pregnant women in real-world studies: a systematic review and meta-analysis. Vaccines (Basel) 2022;10:246. 10.3390/vaccines10020246 35214704 PMC8879911

[10] Shafiee A , Kohandel Gargari O , Teymouri Athar MM , et al . COVID-19 vaccination during pregnancy: a systematic review and meta-analysis. BMC Pregnancy Childbirth 2023;23:45. 10.1186/s12884-023-05374-2 36670389 PMC9853484

[11] Rimmer MP , Teh JJ , Mackenzie SC , et al . The risk of miscarriage following COVID-19 vaccination: a systematic review and meta-analysis. Hum Reprod 2023;38:840–52. 10.1093/humrep/dead036 36794918 PMC10152171

[12] Prasad S , Kalafat E , Blakeway H , et al . Systematic review and meta-analysis of the effectiveness and perinatal outcomes of COVID-19 vaccination in pregnancy. Nat Commun 2022;13:2414. 10.1038/s41467-022-30052-w 35538060 PMC9090726

[13] Pratama NR , Wafa IA , Budi DS , et al . mRNA COVID-19 vaccines in pregnancy: a systematic review. PLoS One 2022;17:e0261350. 10.1371/journal.pone.0261350 35108277 PMC8809595

[14] Yap M , Debenham L , Kew T , et al . Clinical manifestations, prevalence, risk factors, outcomes, transmission, diagnosis and treatment of COVID-19 in pregnancy and postpartum: a living systematic review protocol. BMJ Open 2020;10:e041868. 10.1136/bmjopen-2020-041868 PMC7712931 33268430

[15] Tulane University, Institute for Clinical Effectiveness and Health Policy, London School of Hygiene and Tropical Medicine . COVID-19 vaccines for pregnant persons: a living systematic review and meta-analysis. n.d. Available: safeinpregnancy.org/lsr/

[16] Sterne JA , Hernán MA , Reeves BC , et al . ROBINS-I: a tool for assessing risk of bias in non-randomised studies of interventions. BMJ 2016;355:i4919. 10.1136/bmj.i4919 27733354 PMC5062054

[17] Carlsen EØ , Magnus MC , Oakley L , et al . Association of COVID-19 vaccination during pregnancy with incidence of SARS-CoV-2 infection in infants. JAMA Intern Med 2022;182:825–31. 10.1001/jamainternmed.2022.2442 35648413 PMC9161123

[18] Danino D , Ashkenazi-Hoffnung L , Diaz A , et al . Effectiveness of BNT162b2 vaccination during pregnancy in preventing hospitalization for severe acute respiratory syndrome coronavirus 2 in infants. J Pediatr 2023;254:48–53. 10.1016/j.jpeds.2022.09.059 36252864 PMC9568274

[19] Guedalia J , Lipschuetz M , Calderon-Margalit R , et al . Effectiveness of a third BNT162b2 mRNA COVID-19 vaccination during pregnancy: a national observational study in Israel. Nat Commun 2022;13:6961. 10.1038/s41467-022-34605-x 36379951 PMC9664047

[20] Schrag SJ , Verani JR , Dixon BE , et al . Estimation of COVID-19 mRNA vaccine effectiveness against medically attended COVID-19 in pregnancy during periods of Delta and Omicron variant predominance in the United States. JAMA Netw Open 2022;5:e2233273. 10.1001/jamanetworkopen.2022.33273 36156146 PMC9513651

[21] Villar J , Soto Conti CP , Gunier RB , et al . Pregnancy outcomes and vaccine effectiveness during the period of Omicron as the variant of concern, INTERCOVID-2022: a multinational, observational study. The Lancet 2023;401:447–57. 10.1016/S0140-6736(22)02467-9 PMC9910845 36669520

[22] Fell DB , Dhinsa T , Alton GD , et al . Association of COVID-19 vaccination in pregnancy with adverse peripartum outcomes. JAMA 2022;327:1478–87. 10.1001/jama.2022.4255 35323842 PMC8949767

[23] Goldshtein I , Steinberg DM , Kuint J , et al . Association of BNT162b2 COVID-19 vaccination during pregnancy with neonatal and early infant outcomes. JAMA Pediatr 2022;176:470–7. 10.1001/jamapediatrics.2022.0001 35142809 PMC8832306

[24] Ibroci E , Liu X , Lieb W , et al . Impact of prenatal COVID-19 vaccination on delivery and neonatal outcomes: results from a New York City cohort. Vaccine 2023;41:649–56. 10.1016/j.vaccine.2022.09.095 36526507 PMC9749885

[25] Boelig RC , Aghai ZH , Chaudhury S , et al . Impact of COVID-19 disease and COVID-19 vaccination on maternal or fetal inflammatory response, placental pathology, and perinatal outcomes. Am J Obstet Gynecol 2022;227:652–6. 10.1016/j.ajog.2022.05.049 35640704 PMC9144840

[26] Cao M , Wu Y , Lin Y , et al . Inactivated COVID-19 vaccine did not undermine live birth and neonatal outcomes of women with frozen-thawed embryo transfer. Hum Reprod 2022;37:2942–51. 10.1093/humrep/deac220 36200874 PMC9619751

[27] Citu IM , Citu C , Gorun F , et al . The risk of spontaneous abortion does not increase following first trimester mRNA COVID-19 vaccination. J Clin Med 2022;11:1698. 10.3390/jcm11061698 35330023 PMC8955378

[28] Dick A , Rosenbloom JI , Gutman-Ido E , et al . Safety of SARS-CoV-2 vaccination during pregnancy- obstetric outcomes from a large cohort study. BMC Pregnancy Childbirth 2022;22. 10.1186/s12884-022-04505-5 PMC8884102 35227233

[29] Dick A , Rosenbloom JI , Karavani G , et al . Safety of third SARS-CoV-2 vaccine (booster dose) during pregnancy. Am J Obstet Gynecol MFM 2022;4:100637. 10.1016/j.ajogmf.2022.100637 35398583 PMC8988438

[30] Hui L , Marzan MB , Rolnik DL , et al . Reductions in stillbirths and preterm birth in COVID-19–vaccinated women: a multicenter cohort study of vaccination uptake and perinatal outcomes. Am J Obstet Gynecol 2023;228:585. 10.1016/j.ajog.2022.10.040 PMC9632261 36336084

[31] Magnus MC , Örtqvist AK , Dahlqwist E , et al . Association of SARS-CoV-2 vaccination during pregnancy with pregnancy outcomes. JAMA 2022;327:1469–77. 10.1001/jama.2022.3271 35323851 PMC8949721

[32] Peretz-Machluf R , Hirsh-Yechezkel G , Zaslavsky-Paltiel I , et al . Obstetric and neonatal outcomes following COVID-19 vaccination in pregnancy. JCM 2022;11:2540. 10.3390/jcm11092540 35566665 PMC9105434

[33] Rottenstreich M , Sela H , Rotem R , et al . Uptake and outcomes of COVID-19 vaccination during the third trimester of pregnancy: a multicenter study. AJOG 2022;226:S401–2. 10.1016/j.ajog.2021.11.670 PMC8652528 34554630

[34] Wainstock T , Yoles I , Sergienko R , et al . Prenatal maternal COVID-19 vaccination and pregnancy outcomes. Vaccine 2021;39:6037–40. 10.1016/j.vaccine.2021.09.012 34531079 PMC8421099

[35] Örtqvist AK , Dahlqwist E , Magnus MC , et al . COVID-19 vaccination in pregnant women in Sweden and Norway. Vaccine 2022;40:4686–92. 10.1016/j.vaccine.2022.06.083 35842337 PMC9273610

[36] Blakeway H , Prasad S , Kalafat E , et al . COVID-19 vaccination during pregnancy: coverage and safety. Am J Obstet Gynecol 2022;226:236. 10.1016/j.ajog.2021.08.007 PMC8352848 34389291

[37] Stock* S , Calvert C , Carruthers J , et al . Early pregnancy outcomes following COVID-19 vaccination and SARS-coV-2 infection: a national population-based matched cohort study. In Review [Preprint]. 10.21203/rs.3.rs-1955486/v1 PMC9574832 36253471

[38] Bleicher I , Kadour-Peero E , Sagi-Dain L , et al . Early exploration of COVID-19 vaccination safety and effectiveness during pregnancy: interim descriptive data from a prospective observational study. Vaccine 2021;39:6535–8. 10.1016/j.vaccine.2021.09.043 34600749 PMC8463327

[39] Goldshtein I , Nevo D , Steinberg DM , et al . Association between BNT162b2 vaccination and incidence of SARS-Cov-2 infection in pregnant women. JAMA 2021;326:728–35. 10.1001/jama.2021.11035 34251417 PMC8276131

[40] de Freitas Paganoti C , Alkmin da Costa R , Papageorghiou AT , et al . COVID-19 vaccines confer protection in hospitalized pregnant and postpartum women with severe COVID-19: a retrospective cohort study. Vaccines (Basel) 2022;10:749. 10.3390/vaccines10050749 35632505 PMC9146232

[41] Theiler RN , Wick M , Mehta R , et al . Pregnancy and birth outcomes after SARS-CoV-2 vaccination in pregnancy. Am J Obstet Gynecol MFM 2021;3:100467. 10.1016/j.ajogmf.2021.100467 34425297 PMC8378017

[42] Piekos SN , Hwang YM , Roper RT , et al . Effect of COVID-19 vaccination and booster on maternal–fetal outcomes: a retrospective cohort study. Lancet Digit Health 2023;5:e594–606. 10.1016/S2589-7500(23)00093-6 37537121 PMC10473855

[43] Kim H , Kim H-S , Kim HM , et al . Impact of vaccination and the Omicron variant on COVID-19 severity in pregnant women. Am J Infect Control 2023;51:351–3. 10.1016/j.ajic.2022.07.023 35921943 PMC9339152

[44] Halasa NB , Olson SM , Staat MA , et al . Maternal vaccination and risk of hospitalization for COVID-19 among infants. N Engl J Med 2022;387:109–19. 10.1056/NEJMoa2204399 35731908 PMC9342588

[45] Sekkarie A , Woodruff R , Whitaker M , et al . Characteristics and treatment of hospitalized pregnant women with COVID-19. Am J Obstet Gynecol MFM 2022;4:100715. 10.1016/j.ajogmf.2022.100715 35970493 PMC9371979

[46] Citu IM , Citu C , Gorun F , et al . Immunogenicity following administration of BNT162b2 and Ad26.Cov2.S COVID-19 vaccines in the pregnant population during the third trimester. Viruses 2022;14:307. 10.3390/v14020307 35215900 PMC8878278

[47] Lipkind HS , Vazquez-Benitez G , DeSilva M , et al . Receipt of COVID-19 vaccine during pregnancy and Preterm or small-for-gestational-age at birth — eight integrated health care organizations, United States. MMWR Morb Mortal Wkly Rep 2020;71:26–30. 10.15585/mmwr.mm7101e1 PMC8735559 34990445

[48] Juttukonda LJ , Wachman EM , Boateng J , et al . The impact of maternal SARS‐Cov‐2 vaccination and first trimester infection on Feto‐Maternal immune responses. Am J Reprod Immunol 2022;88:e13625. 10.1111/aji.13625 36123778 PMC9538740

[49] Kashani-Ligumsky L , Lopian M , Cohen R , et al . Titers of SARS CoV-2 antibodies in cord blood of neonates whose mothers contracted SARS CoV-2 (COVID-19) during pregnancy and in those whose mothers were vaccinated with mRNA to SARS CoV-2 during pregnancy. J Perinatol 2021;41:2621–4. 10.1038/s41372-021-01216-1 34564695 PMC8475451

[50] Kugelman N , Riskin A , Kedar R , et al . Safety of COVID‐19 vaccination in pregnant women: a study of the adverse perinatal outcomes. Int J Gynaecol Obstet 2023;161:298–302. 10.1002/ijgo.14599 36452977 PMC9877750

[51] Li M , Hao J , Jiang T , et al . Maternal and neonatal safety of COVID‐19 vaccination during the peri‐pregnancy period: a prospective study. J Med Virol 2023;95:e28378. 10.1002/jmv.28378 36478410 PMC9878102

[52] Lis-Kuberka J , Berghausen-Mazur M , Orczyk-Pawiłowicz M . Attitude and level of COVID-19 vaccination among women in reproductive age during the fourth pandemic wave: a cross-sectional study in Poland. Int J Environ Res Public Health 2022;19:6872. 10.3390/ijerph19116872 35682455 PMC9180577

[53] Smithgall MC , Murphy EA , Schatz-Siemers N , et al . Placental pathology in women vaccinated and unvaccinated against SARS-CoV-2. Am J Obstet Gynecol 2022;227:782–4. 10.1016/j.ajog.2022.06.039 35777431 PMC9236917

[54] Shanes ED , Otero S , Mithal LB , et al . Severe acute respiratory syndrome Coronavirus 2 (SARS-Cov-2) vaccination in pregnancy. Obstetrics & Gynecology 2021;138:281–3. 10.1097/AOG.0000000000004457 33975329 PMC8288194

[55] Rottenstreich M , Rotem R , Wiener-Well Y , et al . Covid-19 third vaccination during pregnancy: maternal and neonatal outcomes—a retrospective study. Arch Gynecol Obstet 2023;308:1197–205. 10.1007/s00404-022-06786-9 36155854 PMC9513010

[56] Wang Y , Ren X , Wang Z , et al . Receipt of Inactivated COVID-19 vaccine had no adverse influence on embryo implantation, clinical pregnancy and Miscarriage in early pregnancy. Sci China Life Sci 2022;65:2332–4. 10.1007/s11427-022-2133-3 35696015 PMC9189441

[57] Beharier O , Plitman Mayo R , Raz T , et al . Efficient maternal to neonatal transfer of antibodies against SARS-CoV-2 and BNT162b2 mRNA COVID-19 vaccine. J Clin Invest 2021;131:e150319. 10.1172/JCI150319 34596052 PMC8483743

[58] Fell DB , Dimanlig-Cruz S , Regan AK , et al . Risk of Preterm birth, small for gestational age at birth, and Stillbirth after COVID-19 vaccination during pregnancy: population based retrospective cohort study. BMJ 2022;378:e071416. 10.1136/bmj-2022-071416 35977737 PMC9382031

[59] Mayo RP , Raz T , Ben DB , et al . Waning of the humoral response to SARS-Cov-2 in pregnancy is variant-dependent. medRxiv 2021. 10.1101/2021.11.03.21265478

[60] UK Health Security Agency . COVID-19 vaccine surveillance report; 2022.

[61] Gray KJ , Bordt EA , Atyeo C , et al . Coronavirus disease 2019 vaccine response in pregnant and lactating women: a cohort study. Am J Obstet Gynecol 2021;225:303. 10.1016/j.ajog.2021.03.023 PMC7997025 33775692

[62] Kachikis A , Englund JA , Singleton M , et al . Short-term reactions among pregnant and lactating individuals in the first wave of the COVID-19 vaccine rollout. JAMA Netw Open 2021;4:e2121310. 10.1001/jamanetworkopen.2021.21310 34402893 PMC8371565

[63] Kadali RAK , Janagama R , Peruru SR , et al . Adverse effects of COVID-19 messenger RNA vaccines among pregnant women: a cross-sectional study on healthcare workers with detailed self-reported symptoms. Am J Obstet Gynecol 2021;225:458–60. 10.1016/j.ajog.2021.06.007 34118200 PMC8189739

[64] Komine-Aizawa S , Haruyama Y , Deguchi M , et al . The vaccination status and adverse effects of COVID ‐19 vaccine among pregnant women in Japan in 2021. J Obstet Gynaecol Res 2022;48:1561–9. 10.1111/jog.15285 35537777 PMC9347631

[65] Mascolo A , di Mauro G , Fraenza F , et al . Maternal, fetal and neonatal outcomes among pregnant women receiving COVID-19 vaccination: the Preg-Co-VAX study. Front Immunol 2022;13:965171. 10.3389/fimmu.2022.965171 36263025 PMC9574088

[66] Montalti M , Guaraldi F , Di Valerio Z , et al . Adherence to and early adverse events of COVID-19 vaccine in a cohort of 600 Italian breastfeeding and pregnant physicians. Hum Vaccin Immunother 2022;18:2106747. 10.1080/21645515.2022.2106747 35944074 PMC9746389

[67] Sadarangani M , Soe P , Shulha HP , et al . Safety of COVID-19 vaccines in pregnancy: a Canadian national vaccine safety (CANVAS) network cohort study. Lancet Infect Dis 2022;22:1553–64. 10.1016/S1473-3099(22)00426-1 35964614 PMC9371587

[68] Shimabukuro TT , Kim SY , Myers TR , et al . Preliminary findings of mRNA Covid-19 vaccine safety in pregnant persons. N Engl J Med 2021;384:2273–82. 10.1056/NEJMoa2104983 33882218 PMC8117969

[69] Toussia-Cohen S , Yinon Y , Peretz-Machluf R , et al . Early adverse events and immune response following second and third COVID-19 vaccination in pregnancy. J Clin Med 2022;11:4720. 10.3390/jcm11164720 36012958 PMC9409660

[70] Voiniušytė A , Černiauskaitė M , Paliulytė V , et al . Vaccination against COVID-19 disease during pregnancy. Acta Med Litu 2022;29:51–7. 10.15388/Amed.2021.29.1.11 36061938 PMC9428649

[71] Zdanowski W , Markiewicz A , Zdanowska N , et al . Tolerability of the BNT162b2 COVID-19 vaccine during pregnancy among Polish Healthcare professionals. Vaccines (Basel) 2022;10:200. 10.3390/vaccines10020200 35214659 PMC8876673

[72] Sourouni M , Braun J , Oelmeier K , et al . Assessment of neonatal cord blood SARS-CoV-2 antibodies after COVID-19 vaccination in pregnancy: a prospective cohort study. Geburtshilfe Frauenheilkd 2022;82:510–6. 10.1055/a-1721-4908 35528187 PMC9076212

[73] Arulappen AL , Danial M , Shanmugam G , et al . A multicenter cohort study on the adverse effects evaluation after messenger RNA COVID-19 vaccination among pregnant healthcare employees in Penang general hospitals. Front Public Health 2022;10:876966. 10.3389/fpubh.2022.876966 35677772 PMC9168536

[74] Ben‐Mayor Bashi T , Amikam U , Ashwal E , et al . The association of maternal SARS‐CoV‐2 vaccination‐to‐delivery interval and the levels of maternal and cord blood antibodies. Intl J Gynecology & Obste 2022;156:436–43. 10.1002/ijgo.14014 PMC9087624 34762739

[75] Blakeway H , Amin-Chowdhury Z , Prasad S , et al . Evaluation of immunogenicity and reactogenicity of COVID ‐19 vaccines in pregnant women. Ultrasound Obstet Gynecol 2022;60:673–80. 10.1002/uog.26050 36318630 PMC9538835

[76] Bookstein Peretz S , Regev N , Novick L , et al . Short‐Term outcome of pregnant women vaccinated with BNT162b2 mRNA COVID ‐19 vaccine. Ultrasound Obstet Gynecol 2021;58:450–6. 10.1002/uog.23729 34198360 PMC8441755

[77] Collier A-RY , McMahan K , Yu J , et al . Immunogenicity of COVID-19 mRNA vaccines in pregnant and lactating women. JAMA 2021;325:2370–80. 10.1001/jama.2021.7563 33983379 PMC8120446

[78] DeSilva M , Haapala J , Vazquez-Benitez G , et al . Evaluation of acute adverse events after COVID-19 vaccination during pregnancy. N Engl J Med 2022;387:187–9. 10.1056/NEJMc2205276 35731916 PMC9258750

[79] Favre G , Maisonneuve E , Pomar L , et al . COVID-19 mRNA vaccine in pregnancy: results of the Swiss COVI-PREG registry, an observational prospective cohort study. Lancet Reg Health Eur 2022;18:100410. 10.1016/j.lanepe.2022.100410 35651954 PMC9148537

[80] Gandhi AP , Thakur JS , Gupta M , et al . COVID-19 vaccination uptake and adverse events following COVID-19 immunization in pregnant women in northern India: a prospective, comparative, cohort study. J Rural Med 2022;17:228–35. 10.2185/jrm.2022-025 36397796 PMC9613372

[81] Rahman MdM , Masum MdHU , Wajed S , et al . A comprehensive review on COVID-19 vaccines: development, effectiveness, adverse effects, distribution and challenges. VirusDis 2022;33:1–22. 10.1007/s13337-022-00755-1 PMC8806010 35127995

[82] Zheng C , Shao W , Chen X , et al . Real-world effectiveness of COVID-19 vaccines: a literature review and meta-analysis. Int J Infect Dis 2022;114:252–60. 10.1016/j.ijid.2021.11.009 34800687 PMC8595975

[83] Dean NE , Hogan JW , Schnitzer ME . Covid-19 vaccine effectiveness and the test-negative design. N Engl J Med 2021;385:1431–3. 10.1056/NEJMe2113151 34496195 PMC8451180

[84] European Centre for Disease Prevention and Control . Overview of the implementation of COVID-19 vaccination strategies and deployment plans in the EU/EEA; 2022.

[85] UK Health Security Agency . COVID-19 vaccination: a guide on pregnancy and breastfeeding. 2020. Available: https://www.gov.uk/government/publications/covid-19-vaccination-women-of-childbearing-age-currently-pregnant-planning-a-pregnancy-or-breastfeeding/covid-19-vaccination-a-guide-on-pregnancy-and-breastfeeding

[86] American College of Obstetricians and Gynecologists . COVID-19 vaccination considerations for obstetric–gynecologic care. 2020. Available: https://www.acog.org/clinical/clinical-guidance/practice-advisory/articles/2020/12/covid-19-vaccination-considerations-for-obstetric-gynecologic-care?report=reader

[87] World Health Organization . WHO SAGE roadmap on uses of COVID-19 vaccines in the context of OMICRON and substantial population immunity. 2023. Available: https://www.who.int/publications/i/item/WHO-2019-nCoV-Vaccines-SAGE-Roadmap

[88] Fragkou PC , Dimopoulou D . Serious complications of COVID-19 vaccines: a mini-review. Metabol Open 2021;12:100145. 10.1016/j.metop.2021.100145 34746732 PMC8556676

[89] World Health Organization . Global COVID-19 vaccination strategy in a changing world. 2022. Available: https://www.who.int/publications/m/item/global-covid-19-vaccination-strategy-in-a-changing-world--july-2022-update

[90] World Health Organization . WHO SAGE roadmap for prioritizing uses of COVID-19 vaccines. 2023. Available: https://www.who.int/publications/i/item/WHO-2019-nCoV-Vaccines-SAGE-Prioritization-2023.1

[91] World Health Organization . HRP annual report 2022. Geneva World Health Organization; 2023.

[92] Murewanhema G . Vaccination hesitancy among women of reproductive age in resource-challenged settings: a cause for public health concern. Pan Afr Med J 2021;38:336. 10.11604/pamj.2021.38.336.28953 34285758 PMC8265245

