# Supplementary material for: Effectiveness and safety of COVID-19 vaccines on maternal and perinatal outcomes: a systematic review and meta-analysis
Source: BMJ Glob Health. 2024 Apr 4;9(4):e014247. doi: 10.1136/bmjgh-2023-014247 (PMC11002410; doi:10.1136/bmjgh-2023-014247)
Supplement: Supplementary data [file bmjgh-2023-014247supp002.pdf]

Appendix 2. Details of search strategies used to include studies in the living systematic review on COVID-19 in pregnant and recently pregnant women

1. Cochrane Gynaecology and Fertility

Pubmed

Item	Term
1	pregnancy/
2	pregnan*.tw.
3	neonatal.tw.
4	perinatal.tw.
5	mothers/.
6	mother.tw.
7	maternal.tw.
8	obstetric.tw.
9	infant, newborn/
10	infant.tw.
11	newborn.tw.
12	child*.tw.
13	or/1-12
14	COVID-19.tw.
15	COVID-2019.tw.
16	severe acute respiratory syndrome coronavirus 2.tw.
17	2019-nCoV.tw.
18	SARS-CoV-2.tw.
19	2019nCoV.tw
20	or/14-19
21	coronavirus.tw.
22	2019/12.pd
23	2020.pd.
24	or/22-23
25	21 and 24
24	or/20-25
25	13 and 24

Google Scholar and Google
Using the following text words (pregnancy OR neonatal OR perinatal OR maternal OR obstetric OR newborn) AND (COVID-19 or SARS-Cov-2)

2. EPPI Centre

The MEDLINE search strategy is the OVID Expert Search as developed by Wolters Kluwer and available at http://tools.ovid.com/coronavirus/

MEDLINE search strategy

1 exp Coronavirus/
2 exp Coronavirus Infections/
3 (coronavirus* or corona virus* or OC43 or NL63 or 229E or HKU1 or HCoV* or ncov* or covid* or sars-cov* or sarscov* or Sars-coronavirus* or Severe Acute Respiratory Syndrome Coronavirus*).mp.
4 (or/1-3) and ((20191* or 202*).dp. or 20190101:20301231.(ep).)
5 4 not (SARS or SARS-CoV or MERS or MERS-CoV or Middle East respiratory syndrome or camel* or dromedar* or equine or coronary or coronal or covidence* or covidien or influenza virus or HIV or bovine or calves or TGEV or feline or porcine or BCoV or PED or PEDV or PDCoV or FIPV or FCoV or SADS-CoV or canine or CCov or zoonotic or avian influenza or H1N1 or H5N1 or H5N6 or IBV or murine corona*).mp.
6 ((pneumonia or covid* or coronavirus* or corona virus* or ncov* or 2019-ncov or sars*).mp. or exp pneumonia/) and Wuhan.mp.
7 (2019-ncov or ncov19 or ncov-19 or 2019-novel CoV or sars-cov2 or sars-cov-2 or sarscov2 or sarscov-2 or Sars-coronavirus2 or Sars-coronavirus-2 or SARS-like coronavirus* or coronavirus-19 or covid19 or covid-19 or covid 2019 or ((novel or new or nouveau) adj2 (CoV on nCoV or covid or coronavirus* or corona virus or Pandemi*2)) or ((covid or covid19 or covid-19) and pandemic*2) or (coronavirus* and pneumonia)).mp.
8 COVID-19.rx,px,ox. or severe acute respiratory syndrome coronavirus 2.os.
9 ("32240632" or "32236488" or "32268021" or "32267941" or "32169616" or "32267649" or "32267499" or "32267344" or "32248853" or "32246156" or "32243118" or "32240583" or "32237674" or "32234725" or "32173381" or "32227595" or "32185863" or "32221979" or "32213260" or "32205350" or "32202721" or "32197097" or "32196032" or "32188729" or "32176889" or "32088947" or "32277065" or "32273472" or "32273444" or "32145185" or "31917786" or "32267384" or "32265186" or "32253187" or "32265567" or "32231286" or "32105468" or "32179788" or "32152361" or "32152148" or "32140676" or "32053580" or "32029604" or "32127714" or "32047315" or "32020111" or "32267950" or "32249952" or "32172715").ui.
10 or/6-9
11 5 or 10

The Embase search strategy as at 21st April 2020

1 exp Coronavirus Infections/
2 exp coronavirinae/
3 (coronavirus* or corona virus* or OC43 or NL63 or 229E or HKU1 or HCoV* or ncov* or covid* or sars-cov* or sarscov* or Sars-coronavirus* or Severe Acute Respiratory Syndrome Coronavirus*).mp.
4 or/1-3
5 4 not (SARS or SARS-CoV or MERS or MERS-CoV or Middle East respiratory syndrome or camel* or dromedar* or equine or coronary or coronal or covidence* or covidien or

influenza virus or HIV or bovine or calves or TGEV or feline or porcine or BCoV or PED or PEDV or PDCoV or FIPV or FCoV or SADS-CoV or canine or CCov or zoonotic or avian influenza or H1N1 or H5N1 or H5N6 or IBV or murine corona*).mp.
6 ((pneumonia or covid* or coronavirus* or corona virus* or ncov* or 2019-ncov or sars*).mp. or exp pneumonia/) and Wuhan.mp.
7 (2019-ncov or ncov19 or ncov-19 or 2019-novel CoV or sars-cov2 or sars-cov-2 or sarscov2 or sarscov-2 or Sars-coronavirus2 or Sars-coronavirus-2 or SARS-like coronavirus* or coronavirus-19 or covid19 or covid-19 or covid 2019 or ((novel or new or nouveau) adj2 (CoV on nCoV or covid or coronavirus* or corona virus or Pandemi*2)) or ((covid or covid19 or covid-19) and pandemic*2) or (coronavirus* and pneumonia)).mp.
8 6 or 7
9 5 or 8

3. WHO COVID-19 database

The WHO COVID-19 database contained articles on the novel coronavirus from the following sources:

- Web of Science
- Oxford Academic Journals
- Pubmed NIH
- Ishiyaku
- J Stage
- Cinii articles
- Ichushi Web – JAMAS
- Science Direct
- Wiley Online Journals
- JAMA Network
- British Medical Journal
- Mary Ann Liebert
- New England Journal of Medicine
- Sage Publications
- Taylor and Francis Online
- Springer Link
- Biomed Central
- MDPI
- ASM
- PLOS
- The Lancet
- Cell Press
- Cell Press Search Interface
- EMBASE
- KoreaMed

Fernández-García S, *et al. BMJ Glob Health* 2024; 9:e014247. doi: 10.1136/bmjgh-2023-014247

- Global Index Medics
- MMWR
- Epidemiology and Health
- American Chemical Society
- Eurosurvellance
- Cambridge Press
- LWW
- Airiti
- JIMR
- Emerging Infectious Diseases
- Osong Public Health & Research Perspectives
- BASE Bielefeld
- LitCOVID

An additional step using the following search terms was added to the WHO search from 12th May 2020

tw:(newborn* OR mother* OR bab* OR wom* OR pregnan* OR postpart* OR neonat* OR fetus OR fetal OR newborn OR mother OR bab*)
