# Supplementary material for: Effectiveness and safety of COVID-19 vaccines on maternal and perinatal outcomes: a systematic review and meta-analysis
Source: BMJ Glob Health. 2024 Apr 4;9(4):e014247. doi: 10.1136/bmjgh-2023-014247 (PMC11002410; doi:10.1136/bmjgh-2023-014247)
Supplement: Supplementary data [file bmjgh-2023-014247supp003.pdf]

Appendix 3. Characteristics of included studies

**First author, year of publication**	**Study design**	**Study setting: country and hospital**<br><br>**Collection period**	**Adjustement variables**	**Inclusion and exclusion criteria**	**Population exposed to covid-19 vaccine**	**Vaccine platform**<br><br>**Vaccine product**<br><br>**Doses (no. women vaccinated)**	**Time of vaccination (trimester)**	**Reported outcomes of interest**
Arulappen AL, 2022	Retrospective cohort	Malaysia<br><br>6 General hospitals in the state of Penang<br><br>March 2021 -	NA	*Inclusion*<br>All pregnant employees who consented to take the mRNA vaccine.<br><br>*Exclusion*<br>Vaccinated pregnant employees who refused to give consent to participate in the study	121	mRNA<br><br>Pfizer-BioNTech<br><br>One dose (121)<br>Two doses (121)	During pregnancy (1st, 2nd, 3rd)	Headache<br>Myalgia<br>Fatigue

Bashi TBM, 2021	Prospective cohort	Israel<br><br>University affiliated tertiary medical center in Tel Aviv<br><br>December 2020 – March 2021	NA	*Inclusion*<br>All women who received the mRNA COVID-19 vaccine during pregnancy who had not prior COVID-19 infection.<br><br>*Exclusion*<br>Unverified timing of vaccination, prior or active infection with covid-19 and refusal to sign informed consent form.	58	mRNA<br><br>Pfizer-BioNTech<br><br>One dose (19)<br>Two doses (39)	During pregnancy (3rd)	Pain at injection site<br>Headache<br>Myalgia<br>Fatigue<br>Fever
Beharier O, 2021	Prospective cohort	Israel<br><br>8 medical centres in Israel<br><br>January 2021 – March 2021	NA	*Inclusion*<br>Pregnant women at an age of 18 years or older and a willingness to participate and provide	92	mRNA<br><br>Pfizer-BioNTech<br><br>Non specified	During pregnancy (Non specified)	Preterm birth <37 weeks<br><br>NICU admission

				informed consent<br><br>*Exclusion*<br>Pregnant women with active maternal COVID-19 disease at delivery				
Blakeway H, 2021	Retrospective cohort	United Kingdom<br><br>St George's University Hospitals<br><br>March 2020 – July 2021	Non specified	*Inclusion*<br>All pregnant women with known vaccination status and complete maternal and foetal outcome data<br><br>*Exclusion*<br>Women who were vaccinated entirely (i.e., all doses) before pregnancy or after birth or	140	mRNA/Viral vector<br><br>Pfizer-BioNTech<br>Moderna<br>AZD<br><br>One dose (114)<br>Two doses (26)	During pregnancy (2nd, 3rd)	Maternal covid infection<br>Severe covid disease<br><br>Caesarean section<br>NICU admission<br>Stillbirth<br>Gestational diabetes<br>Postpartum haemorrhage<br>Small for gestational age

				women who had pregnancies complicated by foetal aneuploidy or genetic syndromes.				
Blakeway H, 2022	Prospective cohort	UK<br><br>April 2021 – September 2021	NA	*Exclusion*<br>Women who did not return the questionnaire.	67	mRNA/Viral vector<br><br>Pfizer-BioNTech<br>Moderna<br>AZD<br><br>One dose (67)<br>Two doses (67)	During pregnancy (1st, 2nd, 3rd)	Headache<br>Fever
Bleicher I, 2021	Prospective cohort	Israel<br><br>Online questionnaire<br><br>January 2021 – February 2021	NA	*Inclusion*<br>All pregnant women that properly filled out the data in questionnaire (valid e-mail address, ID number that	202	mRNA<br><br>Pfizer-BioNTech<br><br>One dose (68)<br>Two doses (124)	During pregnancy (1st, 2nd, 3rd)	Maternal covid infection<br>Miscarriage<br><br>Headache<br>Fever

				matches the information in both questionnaires and answering all questions until the form is submitted)<br><br>*Exclusion*<br>Registries that were invalid or incompatible with the demands (for example: invalid e-mail address, wrong registration of last menstrual period that could not be correct				
Boelig RC, 2022	Retrospective cohort	USA<br><br>Thomas Jefferson University Hospital	Age<br>BMI<br>Ethnicity<br>Diabetes<br>Chronic hypertension	*Inclusion*<br><br><br>*Exclusion*<br>Pregnant patients with both COVID-	49	mRNA<br><br>Non specified<br><br>Non specified	During pregnancy (non specified)	Preterm birth <37 weeks<br>Hypertensive disorder in pregnancy

		March 2020 – July 2021	Prior full-term delivery<br>Prior preterm delivery	19 disease and vaccination				
Bookstein-Peretz S, 2021	Case- control study	Israel<br><br>January 2021-February 2021	NA	*Inclusion*<br>Pregnant women who were vaccinated By a 2-dose regimen of BNT162b2 vaccine between 2-40weeks of gestation and were recruited via social media publications<br><br>*Exclusion*<br>Pregnant women who gave birth or had an abortion before the second	390	mRNA<br><br>Pfizer-BioNTech<br><br>One dose (390)<br>Two doses (390)	During pregnancy (1st, 2nd, 3rd)	Pain at injection site<br>Headache<br>Myalgia<br>Fatigue<br>Fever

				dose of vaccine				
Butt AA, 2021	Cohort	Qatar<br><br>Hamad Medical Corporation<br><br>December 2020 – May 2021	10 years age group Nationality Gestational age	*Exclusion* Women with less than 14 days of follow-up after the second dose, those with a single dose, those with prior SARS-CoV-2 infection and those with pregnancy onset after vaccination.	1053	mRNA<br><br>Pfizer-BioNTech Moderna<br><br>Two doses (407)	During pregnancy (1st, 2nd)	Maternal covid infection
	Test negative case control			*Inclusion* All confirmed pregnant women who presented to Hamad Medical Corporation<br><br>*Exclusion* Women who were tested for		mRNA<br><br>Pfizer-BioNTech Moderna<br><br>Two doses (103)		

				SARS-CoV-2 by RT-PCR on a nasopharyngeal swab prior to pregnancy and those who had no SARS-CoV-2 testing done between December 20, 2020 and May 30, 2021, as well as those who had at least one dose of vaccination before pregnancy onset.				
Cao M, 2022	Retrospective cohort	China<br><br>Guangzhou Medical University<br><br>March 2021 – September 2021	Age<br>Infertility duration<br>Number of COS cicles<br>Protocols of COS<br>Endometrial preparation protocol	*Inclusion*<br>Women with confirmed vaccination status from public health surveillance system record of their smartphone	502	Inactivated virus<br><br>Sinovac-CoronaVac<br>Sinopharm BIBP<br><br>One dose (30)	Before pregnancy	Miscarriage

			Number of embryos transferred<br>Number of top-quality embryos	app, the first or second cycle of frozen-thawed embryos transferred, women in vaccinated group had embryos frozen prior to the exposure to Covid-19 vaccines, and women aged 20-40 years old<br><br>*Exclusion*<br>Women with three or more cycles of controlled ovarian stimulation, women with repeated spontaneous miscarriage, women with		Two doses (472)		

				repeated implantation failure, cycles with surgically obtained sperms, cycles with sperm donor, and infertile couples with severe systemic disease which might reduce conception chance				
Carlsen EO, 2022	Retrospective cohort	Norway<br><br>Medical Birth Registry of Norway<br><br>September 2021 – February 2022	Age<br>Parity<br>Education<br>Country of birth<br>Country of residence	*Inclusion*<br>All live births in Norway between 1 September 2021 and 28 February 2022.<br><br>*Exclusion*<br>Mother and infants with no permanent national	14312	mRNA<br><br>Non specified<br><br>Two doses (8915)<br>Three doses (824)	During pregnancy (2nd, 3rd)	Maternal covid infection<br>Preterm birth <37 weeks

				identification number.				
Citu IM, 2022	Prospective cohort	Romania<br><br>Timisoara Municipality Emergency Hospital<br><br>May 2021 – December 2021	NA	*Inclusion*	227	mRNA/Viral vector<br><br>Pfizer-BioNTech<br>Janssen<br><br>One dose (58)<br>Two doses (115)	During pregnancy (3rd)	Gestational diabetes<br>Hypertensive disorder in pregnancy<br>Caesarean section<br>Preterm birth <37 weeks<br>Postpartum haemorrhage<br>Abnormal apgar 5<br>Small for gestational age<br><br>Pain at injection site<br>Myalgia<br>Headache<br>Fever<br>Fatigue
Citu IM (1) 2022	Retrospective cohort	Romania<br><br>Obstetrics and gynecology	Age (> 35 years)<br>Overweight status (>25)	*Inclusion*<br>All pregnancies in mothers older	927	mRNA<br><br>Pfizer-BioNTech	During pregnancy (1st)	Miscarriage

		clinic of Timisoara Municipal emergency hospital<br><br>January 2020 – January 2022	Chronic conditions<br>Previous SARS-CoV-2 infection<br>Smoker<br>Abnormal uterine or cervical anatomy<br>Previous miscarriage<br>Assisted reproductive techniques<br>Vaccine type<br>Number of doses	than 18 years, evaluated from the start of their first trimester during the study period in the clinic.<br>Only pregnant women vaccinated with BNT162b2 or Moderna mRNA-1273.<br><br>*Exclusion*<br>Patients who did not provide consent were excluded		Moderna<br><br>Two doses (927)		
Collier AY 2021	Prospective cohort	Israel<br><br>Beth Israel Deaconess Medical center	NA	*Inclusion*<br>Women 18 years or older who had received a covid-19 vaccine<br><br>*Exclusion*	30	mRNA<br><br>Pfizer-BioNTech<br>Moderna<br><br>One dose (30)	During pregnancy (1st, 2nd, 3rd)	Fever

		December 2020 – March 2021				Two doses (29)		
COVID-NET, 2021	Retrospective cohort	USA<br><br>Nk<br><br>January 2021 – November 2021	NA	*Inclusion*	11	mRNA/Viral vector<br><br>Pfizer-BioNTech<br>Moderna<br>Janssen<br><br>Non specified	During pregnancy (non specified)	Severe covid disease<br><br>Miscarriage<br>Caesarean section<br>Maternal death<br>Preterm birth <37 weeks<br>Stillbirth
Dagan N, 2021	Prospective cohort	Israel<br><br>Clalit Health Services (CHS)<br><br>December 2020 – June 2021	Age<br>Trimester of pregnancy<br>Geostatistical living area<br>Population sector<br>Count of influenza vaccination in the last 5 years<br>Existence of at least one CDC and prevention	*Inclusion*<br>All pregnant women aged 16 years or older, with continuous membership in CHS<br>for 1 complete year, no previous positive SARS-CoV-2 PCR test, no previous SARS-CoV-2	10861	mRNA<br><br>Pfizer-BioNTech<br><br>Two doses (10861)	During pregnancy (1st, 2nd, 3rd)	Maternal covid infection<br>Severe covid disease<br>Maternal hospital admission<br>Maternal death

			risk factor for severe disease (obesity, diabetes, hypertension)	vaccination, not residing in long-term care facilities, no home confinement due to medical reasons, not being a healthcare worker and no interaction with the healthcare system in the previous 2 d<br><br>*Exclusion*<br>Pregnant women with missing data in CHS				
Danino D 2022	Test negative case-control	Israel<br><br>Soroka Medical center, Schneider medical center, Shamir	Ethnicity<br>Prematurity	*Inclusion*<br>Symptomatic infants suspected of SARS-CoV-2 infection by a physician	202	mRNA<br><br>Pfizer-BioNTech<br><br>Two doses (202)	During pregnancy (2nd, 3rd)	Neonatal covid infection<br>Preterm birth

		medical center<br><br>March 2021 – November 2021		*Exclusion*<br>Infants who were tested during screening or were asymptomatic				
DeSilva M, 2022	Retrospective cohort	USA<br><br>Eigth vaccine safety datalink sites<br><br>December 2020 – July 2021	NA	*Inclusion*<br>Pregnant women between 16 and 49 years	45232	mRNA/Viral vector<br><br>Pfizer-BioNTech<br>Janssen<br><br>One dose (12438)<br>Two doses (32794)	During pregnancy (1st, 2nd, 3rd)	Fever<br>Fatigue
Dick A, 2022	Retrospective cohort	Israel<br><br>Hadassah-Hebrew University Center<br><br>December 2020 – July 2021	Age<br>BMI<br>Nulliparity<br>Smoking	*Inclusion*<br>Women with singleton deliveries from December 2020 until July 2021<br><br>*Exclusion*	2305	mRNA<br><br>Pfizer-BioNTech<br><br>Two doses (non specified)	During pregnancy (2nd, 3rd)	Preterm birth <37 weeks<br>Small for gestational age<br>Caesarean section<br>Postpartum haemorrhage<br>Stillbirth

				Women with multiple pregnancy, vaccination prior to pregnancy, COVID-19 infection during or before pregnancy, or unknown timing of vaccination		Three doses (non specified)		Hypertensive disorder in pregnancy<br>Gestational diabetes<br>Abnormal apgar 5
Dick A (1), 2022	Retrospective cohort	Israel<br><br>Nr<br><br>July 2021 – October 2021	Age<br>BMI<br>Nulliparity<br>Gestational diabetes<br>Smoking	*Inclusion*<br>Women with singleton pregnancies who delivered in the period July-October 2021<br><br>*Exclusion*<br>Women with multiple pregnancy, COVID-19 infection during or	2845	mRNA<br><br>Pfizer-BioNTech<br>Moderna<br><br>Two doses (2845)<br>Three doses (294)	During pregnancy (3rd)	Preterm birth <37 weeks<br>Small for gestational age<br>Caesarean section<br>Postpartum haemorrhage<br>Stillbirth<br>Hypertensive disorder in pregnancy<br>Gestational diabetes

				before pregnancy, or unknown timing of vaccination				Abnormal apgar 5
Favre G (2), 2022	Prospective cohort	Switzerland<br><br>COVI-PREG registry<br><br>March 2021 – December 2021	NA	*Inclusion*<br>Pregnant women who received at least one injection of a mRNA vaccine against COVID-19 between one week before their last menstrual period and the end of pregnancy were included in the study.<br><br>*Exclusion*<br>Patients under 18 years old who were not able to consent.	894	mRNA<br><br>Pfizer-BioNTech<br>Moderna<br><br>Two doses (894)	During pregnancy (1st, 2nd, 3rd)	Pain at injection site<br>Fatigue<br>Headache<br>Myalgia<br>Fever

				Women with no information on the date of injection, the occurrence of early adverse events and their description if any or no information about the type of vaccine used				
Fell DB, 2022	Retrospective cohort	Canada<br><br>Better Outcome Registry and Network Ontario Birth Registry<br><br>May 2021 – December 2021	NA	*Inclusion*<br>Completed pregnancies between 1 May and 31 December 2021<br><br>*Exclusion*<br>Births to non-Ontario residents and births from pregnancies conceived less	43099	mRNA/Viral vector<br><br>Pfizer-BioNTech<br>Moderna<br>AZD<br><br>One dose (13416)<br>Two doses (29650)<br>Three doses (33)	During pregnancy (1st, 2nd, 3rd)	Stillbirth<br>Preterm birth < 37 weeks<br>Small for gestational age

				than 42 weeks before the end of the study. Any records with gestational age < 20 weeks and birth weight < 500 gr, or following pregnancy termination				
Fell DB (1), 2022	Retrospective cohort	Canada<br><br>Better Outcome Registry and Network Ontario Birth Registry<br><br>December 2020 – September 2021	NA	*Inclusion*<br>Pregnancies with a birth date or expected due date on or after December 14, 2020 (when the COVID-19 vaccination program began in Ontario)<br><br>*Exclusion*<br>Ongoing pregnancies as of September	22660	mRNA/Viral vector<br><br>Pfizer-BioNTech<br>Moderna<br>AZD<br><br>Non specified	During pregnancy (1st, 2nd, 3rd)	Maternal covid infection<br>Postpartum haemorrhage<br>Caesarean section<br>NICU admission<br>Abnormal apgar 5

				30, 2021, individuals who became pregnant less than 42 weeks before the end of the study period (i.e., those with a last menstrual period after December 9, 2020), records with documented gestational age less than 20 weeks at birth, and pregnancy terminations				
Gandhi AP, 2022	Prospective cohort	India<br><br>July 2021 – October 2021	NA	*Inclusion*<br>Pregnant women registered with the ANC clinics who went for covid-19 vaccination<br><br>*Exclusion*	247	Viral vector<br><br>AZD<br><br>One dose (247)	During pregnancy (non specified)	Fever<br>Pain at injection site

				Women less than 18 years of age and who did not consent to participate in the follow-up				
Goldshtein I, 2021	Retrospective cohort	Israel<br><br>Maccabi Healthcare Services Database<br><br>December 2020 – February 2021	NA	*Inclusion*<br>All pregnant women in the health fund<br><br>*Exclusion*<br>Pregnant women who joined the fund less than 1 year preconception, with any preconception records indicating SARS- CoV-2 infection, and members who were vaccinated pre-pregnancy with the	7530	mRNA<br><br>Pfizer-BioNTech<br><br>Non specified	During pregnancy (non specified)	Maternal covid infection<br>Maternal death<br>Maternal hospital admission<br>Preterm birth <37 weeks<br>Stillbirth<br>Hypertensive disorder in pregnancy

				BNT162b2 mRNA vaccine				
Goldshtein I, 2022	Retrospective cohort	Israel<br><br>Maccabi Healthcare Services Database<br><br>March 2021 – October 2021	NA	*Inclusion*<br>All singleton live births at any time from March 1, 2021, through September 31, 2021<br><br>*Exclusion*<br>Records with no mother-offspring linkage, multiple births, insufficient prior membership time, or missing covariate data	16697	mRNA<br><br>Pfizer-BioNTech<br><br>Non specified	During pregnancy (1st, 2nd, 3rd)	Gestational diabetes<br>Preterm birth <37 weeks<br>Small for gestational age
Gray KJ, 2021	Retrospective cohort	USA<br><br>Questionnaire<br><br>December 2020 –	NA	*Inclusion*<br>Pregnant, lactating and non-pregnant women of reproductive	83	mRNA<br><br>Pfizer-BioNTech<br>Moderna	During pregnancy (1st, 2nd, 3rd)	Pain at injection site<br>Headache<br>Myalgia<br>Fatigue<br>Fever

		February 2021		age (18-45), able to provide informed consent and receiving the covid-19 vaccine<br><br>*Exclusion*		One dose (83)<br>Two doses (77)		
Guedalia J, 2022	Retrospective cohort	Israel<br><br>MOH Database<br><br>August 2021 – March 2022	Age<br>Parity<br>Days of follow-up	*Inclusion*<br>Women who had a documented delivery between August 1, 2021, to March 22, 2022<br><br>*Exclusion*<br>Women who received one vaccine or a fourth boosting dose	60554	mRNA<br><br>Pfizer-BioNTech<br><br>Two doses (60554)	During pregnancy (non specified)	Maternal covid infection<br>Severe covid disease<br>Maternal hospital admission<br>Maternal death
Halasa NB (1), 2022	Test negative case-control	USA<br><br>paediatric hospitals in	NA	*Inclusion*<br>Women who received the first dose	231	mRNA<br><br>Pfizer-BioNTech	During pregnancy (non specified)	Preterm birth <37 weeks<br>NICU admission

		the CDC-funded overcoming COVID-19 network July 2021 – March 2022		before pregnancy and the second dose during pregnancy<br><br>*Exclusion* Women partially vaccinated during pregnancy (I.e., received one dose during pregnancy and no dose before pregnancy) or who had been fully vaccinated before pregnancy or after delivery, women who had been vaccinated less than 14 days before delivery, and		Two doses (231)		

				women who had received a third dose of an mRNA vaccine or had received a non-mRNA vaccine (i.e., Ad26.COV2.S)				
Hui L (1), 2022	Retrospective cohort	Australia<br><br>12 Public Maternity Hospitals in Melbourne<br><br>July 2021 – March 2022	Age<br>BMI<br>Metropolitan vs regional residence<br>Smoking<br>Region of birth<br>Socioeconomic index for postcodes<br>Diabetes<br>Parity<br>Infant sex<br>Gestation at first antenatal visit<br>Need for interpreter	*Inclusion*<br>Births ≥ 20 weeks from all 12 public maternity hospitals in Melbourne from 1st July 2021 to 1st March 2022<br><br>*Exclusion*<br>Births in exclusively private hospitals and planned home births outside of publicly funded	17365	mRNA<br><br>Non specified<br><br>Non specified	Before/during pregnancy	Gestational diabetes<br>Stillbirth<br>Preterm birth <37 weeks<br>NICU admission<br>Abnormal apgar 5<br>Postpartum haemorrhage<br>Caesarean section

				homebirth programs				
Ibroci E, 2022	Prospective cohort	USA<br><br>Mount Sinai Health system<br><br>April 2020-February 2021	NA	*Inclusion*	250	mRNA/Viral vector<br><br>Pfizer-BioNTech<br>Moderna<br>Janssen<br><br>Non specified	During pregnancy (1st, 2nd, 3rd)	Preterm birth <37 weeks<br>NICU admission<br>Caesarean section<br>Small for gestational age
Juttukonda L, 2022	Prospective cohort	USA<br><br>Boston Medical Center<br><br>July 2020 – November 2021	NA	*Inclusion*<br>Age minimum of 18 years, singleton pregnancy, full-term (gestational age ≥ 37 weeks) delivery, and English/Spanish speaking	17	mRNA<br><br>Pfizer-BioNTech<br>Moderna<br><br>Two doses (17)	During pregnancy (non specified)	Hypertensive disorder in pregnancy<br>Preterm birth <37 weeks<br>Caesarean section<br>Gestational diabetes<br>NICU admission
Kachikis AL, 2022	Prospective cohort	USA<br><br>Questionnaire<br><br>January 2021 – March 2021	NA	*Inclusion*	7565	mRNA/Viral vector<br><br>Pfizer-BioNTech<br>Moderna	During pregnancy (1st, 2nd, 3rd)	Fever

						Janssen<br><br>One dose (7565)<br>Two doses (6232)		
Kadali RAK, 2021	Cross-sectional study	USA<br><br>Online questionnaire<br><br>Non specified	NA	*Inclusion*<br>Pregnant women admitted for delivery who consented to sample collection for a biorepository	38	mRNA<br><br>Pfizer-BioNTech<br>Moderna<br><br>One dose (non specified)<br>Two doses (non specified)	During pregnancy (non specified)	Pain at injection site<br>Fatigue<br>Headache<br>Myalgia<br>Fever
Kashani-Ligumsky L, 2021	Retrospective cohort	Israel<br><br>Mayanei Hayeshua medical centre<br><br>February 2021- March 2021	NA	*Inclusion*<br>Women who delivered singleton livebirths<br><br>*Exclusion*<br>Women who contracted SARS-CoV-2 infection prior	29	mRNA<br><br>Pfizer-BioNTech<br><br>Two doses (29)	During pregnancy (3rd)	Ceasarean section<br>NICU admission<br>Preterm birth <37 weeks

				to vaccination and women with single dose				
Kim H, 2022	Retrospective cohort	South Korea<br><br>Kyungpook National University Chilgok Hospital<br><br>November 2020 – March 2022	NA	*Inclusion*<br>All pregnant women admitted to the institution for COVID-infection between November 1, 2020, and March 7, 2022	39	mRNA<br><br>Non specified<br><br>Non specified	During pregnancy (Non specified)	Preterm birth <37 weeks
Komine-Aizawa S, 2022	Retrospective cohort	Japan<br><br>Online questionnaire<br><br>October 2021 – November 2021	NA	*Inclusion*<br>Older than 20 years old or married minors older than 16 years.	5032	mRNA<br><br>Pfizer-BioNTech<br>Moderna<br><br>One dose (5032)<br>Two doses (4587)	During pregnancy (1st, 2nd, 3rd)	Pain at injection site<br>Fever<br>Fatigue<br>Headache
Kugelman N, 2022	Retrospective cohort	Israel<br><br>Carmel Medical Center	NA	*Inclusion*<br>Women with singleton pregnancy	930	mRNA<br><br>Pfizer-BioNTech	During pregnancy (2nd, 3rd)	Caesarean section<br>NICU admission<br>Stillbirth

		February 2021 – July 2021		over 23 weeks of gestation<br><br>*Exclusion*<br>Multiple gestations and those who underwent termination of pregnancy		One dose (51)<br>Two doses (879)		Abnormal Apgar 5
Li M, 2022	Prospective cohort	China<br><br>Beijing Dita Hospital<br><br>March 2021-February 2022	NA	*Inclusion*<br>Pregnant women who inoculated or never inoculated inactivated covid-19 during the peri-pregnancy period.<br>Pregnant women aged between 10 and 45<br><br>*Exclusion*<br>Family history of hereditary diseases in one	93	Inactivated virus<br><br>Sinovac-CoronaVac<br>Sinopharm BIBP<br><br>Non specified	Before/During pregnancy (1st)	Preterm birth <37 weeks<br>Hypertension disorder in pregnancy<br>Postpartum haemorrhage

				or both families of the couple. Women who delivered babies with congenital abnormalities. Three or more spontaneous abortions in the past. Taking drugs that have a definite effect on fetal development during pregnancy. Exposure to toxic substances in early pregnancy. Associated with malignant tumors. Coinfection with hepatitis C, hepatitis D,				

				human immunodeficiency virus, syphilis, toxoplasmosis, rubella or cytomegalovirus.				
Lipkind HS, 2022	Retrospective cohort	USA<br><br>VSD sites in California, Colorado, Minnesota, Oregon, Washington, and Wisconsin (Kaiser Permanente: Colorado, Northern California, Northwest, Southern California, and Washington; Denver Health;	NA	*Inclusion*<br>Singleton live births from eight VSD sites, females aged 16-49 years with estimated pregnancy start during May 17 – October 24, 2020, and expected delivery dates, based on a 40-week gestation, during February 21 – July 31, 2021, and all COVID-19	10064	mRNA/Viral vector<br><br>Pfizer-BioNTech<br>Moderna<br>Janssen<br><br>One dose (2183)<br>Two doses (7881)	During pregnancy (1st, 2nd, 3rd)	Preterm birth <37 weeks<br>Small for gestational age

		HealthPartners; and Marshfield Clinic).<br><br>December 2020 – July 2021		vaccine doses administered from the last menstrual period through 3 days before delivery<br><br>*Exclusion*<br>Vaccines administered within 3 days of delivery				
Lis-Kuberka J, 2022	Cross-sectional	Poland<br><br>Questionnaire<br><br>November 2021 – December 2021	NA	*Inclusion*<br>Women who were pregnant and/or delivered during pandemic of COVID-19<br><br>*Exclusion*<br>Women who did not fully complete the questionnaire or provided unreliable data	796	mRNA/Viral vector<br><br>Pfizer-BioNTech<br>Moderna<br>Janssen<br>AZD<br><br>Non specified	During pregnancy (1st, 2nd, 3rd)	Caesarean section

Magnus MC (3), 2022	Retrospective cohort	Sweden/Norway<br><br>Pregnancy Register in Sweden and The Medical Birth Registry of Norway<br><br>January 2021 – January 2022	Age<br>Gestational age<br>Parity<br>Education<br>Living with a partner<br>Household income<br>Previous positive SARS-CoV-2 test<br>Underlying chronic condition	*Inclusion*<br>All singleton pregnancies ending after 22 completed gestational weeks registered in the Pregnancy Register in Sweden and the Medical Birth Registry of Norway from January 1, 2021, until January 12, 2022 (Sweden), or January 15, 2022 (Norway)<br><br>*Exclusion*<br>Pregnancies ending in multiple births, individuals vaccinated prior to pregnancy, and	28506	mRNA/Viral vector<br><br>Pfizer-BioNTech<br>Moderna<br>AZD<br><br>One dose (6977)<br>Two doses (21529)	During pregnancy (1st, 2nd, 3rd)	Maternal covid infection<br>Abnormal apgar 5<br>Small for gestational age<br>NICU admission<br>Stillbirth<br>Preterm birth <37 weeks

				individuals who received the Johnson & Johnson vaccine				
Mascolo A 2022	Retrospective cohort	Israel<br><br>EV database<br><br>January 2021-December 2021	NA	*Inclusion*<br><br><br>*Excluded*<br>ICSRs with PTs referred to extraction criteria and without AEFI, uncertain information on the vaccine exposure during pregnancy, and sex or age incoherent or unknown	3252	mRNA/Viral vector<br><br>Pfizer-BioNTech<br>Moderna<br>Janssen<br>AZD<br><br>Non specified	During pregnancy (non specified)	Headache<br>Fatigue<br>Myalgia<br>Pain at injection site
Mayo RP, 2021	Prospective cohort	Israel<br><br>8 medical centres in Israel (Hadassah Mount	NA	*Inclusion*<br>Pregnant women admitted for delivery at 8 medical centres, ≥18	125	mRNA<br><br>Pfizer-BioNTech<br><br>Two doses (125)	During pregnancy (2nd, 3rd)	Preterm birth <37 weeks<br>NICU admission

		Scopus, Wolfson, HaEmek, Hillel Yafe, Rabin, Shaare Zedek, Meir, and Sourasky Medical Centers)<br><br>January 2021 – June 2021		years old and willing to provide informed consent<br><br>*Exclusion*<br>Pregnant women with active COVID-19 infection				
Montalti M, 2022	Cross-sectional study	Italy<br><br>Online survey<br><br>– January 2021	NA	*Inclusion*<br>Female members of the Facebook group "Coronavirus, SARS-CoV-2 e COVID-19 gruppo per soli medici" that have been pregnant or breastfeeding for the entire duration of the survey	31	mRNA<br><br>Pfizer-BioNTech<br><br>One dose (31)<br>Two doses (17)	During pregnancy (non specified)	Fever<br>Fatigue<br>Myalgia<br>Headache

				Exclusion				
Ortqvist AK. 2022	Retrospective cohort	Sweden<br>Norway<br>Swedish pregnancy register<br><br>Medical Birth Register in Norway<br><br>May 2021 – May 2022	Age	*Exclusion:*<br>Vaccinated prior pregnancy	69512	mRNA/Viral vector<br><br>Pfizer-BioNTech<br>Moderna<br>AZD<br><br>One dose (9702)<br>Two doses (47699)<br>Booster dose (12111)	Before/during pregnancy (non specified)	Gestational diabetes<br>Hypertensive disorder in pregnancy
Paganoti CDF, 2022	Retrospective cohort	Brazil<br><br>SIVEP-Gripe registry<br><br>May 2021 – November 2021	NA	*Inclusion:*<br>Pregnant or postpartum of childbearing age (10-55 years), COVID confirmed by PCR, SARS CoV-2 or antigen. Exclusion of influenza infection by	200	mRNA/Inactivated virus<br><br>Non specified<br><br>Non specified	During pregnancy (non specified)	Severe covid disease<br>Maternal death

				negative RT-PCR or antigen for influenza. Availability of the outcome (recovery or death) and reliability of vaccination status.<br><br>No exclusion criteria.				
Paixao ES 2022	Test negative case-control	Brazil<br><br>Brazilian Ministry of Health<br><br>January 2021-October 2021	Age<br>Ethnicity<br>Comorbidities<br>Region of residence<br>IBP<br>Time	*Inclusion:* Pregnant women with symptoms suggesting Covid-19, aged between 18 and 49 years in Brazil with a record of a RT-PCR test between March 15, 2021, and October 03, 2021,	2033	Inactivated virus<br><br>Sinovac-CoronaVac<br><br>One dose (995)<br>Two doses (1038)	During pregnancy (non specified)	Maternal covid infection<br>Severe covid disease

				registered in e-SUS Notifica.<br><br>*Exclusion:* Subjects who received any Covid-10 Vaccine: ChAdOx1 nCoV-19 or Ad26.COV2.S (Janssen/Johnson & Johnson) because these are not indicated for pregnant women in Brazil and BNT162b2 numbers of women with complete regimen were too small to allow evaluation given they were included in the				

				Brazilian program.				
Peretz - Machluf R 2022	Retrospective cohort	Israel<br><br>The department of Obstetrics and Gynecology, Chaim Sheba Medical Center<br><br>March 2021 - July 2021	Age<br>Parity<br>Smoking<br>Gestational age at delivery<br>Background conditions (Obesity, hypertensive disorders, diabetes)	*Inclusion*<br>Vaccinated and non-vaccinated pregnant women with singleton pregnancies.<br><br>*Exclusion*<br>Women with prior COVID-19 infection, multiple gestations, and stillbirth	3240	mRNA<br><br>Pfizer-BioNTech<br><br>Non specified	During pregnancy (2nd, 3rd)	Gestational diabetes<br>Hypertensive disorders<br>Small for gestational age<br>Preterm birth <37 weeks<br>Caesarean section<br>Abnormal Apgar 5<br>NICU admission
Piekos SN, 2022	Retrospective cohort	USA<br><br>Providence St Joseph Health Alaska, California, Montana, Oregon, New Mexico, Texas, and Washington	NA	*Inclusion:*<br>18-45 years, with singleton pregnancies Delivery after 20 weeks gestation.<br><br>Positive SARS Cov-2 NAAT test.	34408	mRNA<br><br>Pfizer-BioNTech<br>Moderna<br><br>Two doses (26792)<br>Booster dose (7616)	During pregnancy (1st, 2nd, 3rd)	Maternal covid infection<br>Maternal death<br>Caesarean section<br>Hypertensive disorders<br>Gestational diabetes

		Jan 2021- Jul 2022		Propensity score matching accounting for demographic, lifestyle, geographical and clinical characteristics for negative maternal-fetal outcomes to generate an unvaccinated matched cohort.				
Rottenstreich M, 2022	Retrospective cohort	Israel<br><br>Shaare Zedek Medical Center (SZMC) and the Bikur Holim Medical Center (BHMC)<br><br>January 2021 – April 2021	NA	*Inclusion*<br>All pregnant women admitted for delivery aged 18 years or older, with no documented previous positive SARS-CoV-2	712	mRNA<br><br>Pfizer-BioNTech<br><br>Two doses (712)	During pregnancy (3rd)	Severe covid disease<br>Caesarean section<br>Preterm birth <37 weeks<br>NICU admission<br>Pospartum haemorrhage<br>Abnormal Apgar 5<br>Stillbirth

								Hypertensive disorders<br>Small for gestational age
Rottenstreich M (1), 2022	Retrospective cohort	Israel<br><br><br>August 2021 – December 2021	NA	*Inclusion*<br>All women aged 18 or older, without documented previous positive SARS-CoV-2 PCR test, delivered between August 28 and December 31 2021.<br><br>*Exclusion*<br>Parturients who were previously positive with covid-19 PCR swabs during or before pregnancy.	1720	mRNA<br><br>Pfizer-BioNTech<br><br>Two doses (1094)<br>Booster dose (626)	During pregnancy (non specified)	Caesarean section<br>Postpartum haemorrhage<br>Preterm birth <37 weeks<br>Small for gestational age<br>Stillbirth<br>NICU admission<br>Abnormal Apgar 5

				Women who received only the first dose				
Sadarangani M, 2022	Prospective cohort	Canada<br><br>Seven Canadian provinces and territories<br><br>-November 2021	NA	*Inclusion*<br>Received first dose of an authorized COVID-19 vaccine within the prior seven days; have active email address and telephone number, can communicate in English or French, reside in one of the seven provinces and territories<br><br>*Exclusion*	5597	mRNA<br><br>Pfizer-BioNTech<br>Moderna<br><br>One dose (5597)<br>Two doses (3108)	During pregnancy (1st, 2nd, 3rd)	Myalgia<br>Fever<br>Headache
Schrag SJ, 2022	Test negative case-control	USA<br><br>Network of 306 hospitals and 164	Age<br>Geographic regions<br>Calendar time	*Inclusion:*<br>Aged 18-45 with COVID like illness diagnosis, RT-	1137	mRNA<br><br>Pfizer-BioNTech<br>Moderna	During pregnancy (1st, 2nd, 3rd)	Maternal covid infection

		emergency department and urgent care facilities across 10 US states.<br><br>June 2021 – June 2022	Local virus circulation	PCR for SARS COV-2 during 14 days before through 72 hours after the medical encounter and pregnant at the time of encounter. Acute respiratory illness – respiratory failure, viral or bacterial pneumonia, asthma exacerbation, influenza and viral illness otherwise not specified.<br><br>*Exclusion:* Ad.26.COV2.S Janssen vaccine. Single vaccinated or more than 3		Two doses (721)<br>Three doses (416)		Maternal hosital admission

				doses of mRNA vaccine. Those with less than 14 days between second dose and fewer than 7 days since their third dose.				
Shanes ED (1), 2021	Prospective cohort	USA<br><br>January 2021 - April 2021	NA	*Inclusion*	84	Non specified<br><br>Non specified<br><br>Non specified	During pregnancy (Non specified)	Caesarean delivery
Shimabukuro TT, 2021	Retrospective cohort	USA<br><br>V-safe Surveillance System, V-safe pregnancy registry and VAERS system<br><br>December 2020 –	NA	*Inclusion*<br>Received vaccination during pregnancy or in the periconception period and are 18 years of age or older	16982	mRNA<br><br>Pfizer-BioNTech<br>Moderna<br><br>One dose (16982)<br>Two doses (12273)	During pregnancy (non specified)	Pain at injection site<br>Fatigue<br>Headache<br>Myalgia<br>Fever

		February 2021						
Smithgall MC, 2022	Prospective cohort	USA<br><br>New York city hospital<br><br>April 2020 – July 2021	NA	*Exclusion:* Incomplete vaccine administration<br><br>*Inclusion:* Fully vaccinated women, at least 2 doses of a SARs-COV-2 mRNA vaccine at >2 weeks before delivery, included if they did not have positive anti-N antibodies produced in the setting of infection.	164	mRNA<br><br>Pfizer-BioNTech Moderna<br><br>Two doses (164)	During pregnancy (non specified)	Caesarena section<br>Preterm birth < 37 week<br>Abnormal Apgar 5<br>Small for gestational age
Sourouni M, 2022	Prospective cohort	Germany	NA	*Inclusion* Women given birth at the hospital who	70	mRNA<br><br>Pfizer-BioNTech	During pregnancy (1st, 2nd, 3rd)	Fever<br>Pain at injection site

		University hospital of Münster<br><br>March 2021 – November 2021		were vaccinated during pregnancy		Moderna<br><br>Non specified		
Stock S, 2022	Retrospective study	UK<br><br>COPS cohort<br><br>September 2021- January 2022	Age<br>Gestational age at date of vaccination<br>Deprivation<br>Urban/rural status<br>Clinical vulnerability	*Inclusion:* Vaccinated from 6 weeks before conception to 19 weeks and 6 days gestation for miscarriage and 2+6 weeks for ectopic pregnancy.<br><br>*Exclusion:* Completed pregnancies with unknown pregnancy outcome.	18780	mRNA/Viral vector<br><br>Pfizer-BioNTech<br>Moderna<br>AZD<br><br>One dose (non specified)<br>Two doses (Non specified)<br>Booster dose (Non specified)	Before/During pregnancy (1st, 2nd)	Miscarriage

Theiler RN, 2021	Retrospective cohort	United States of America<br><br>Hospitals within the Mayo Clinic Health System<br><br>December 2021 – April 2021	NA	*Inclusion*<br>All women aged 16-55 years old who delivered between December 10, 2020, and April 19, 2021 at a Mayo Clinic hospital.<br><br>*Exclusion*<br>Minnesota patients who delivered in Minnesota and opted out of use of their medical records for research	140	mRNA/Viral vector<br><br>Pfizer-BioNTech<br>Moderna<br>Janssen<br><br>Non specified	During pregnancy (1st, 2nd, 3rd)	Maternal covid infection<br>Severe covid disease<br>Maternal death<br>Caesarean section<br>Preterm birth <37 weeks<br>NICU admission<br>Abnormal Apgar 5<br>Stillbirth<br>Hypertensive disorders<br>Postpartum haemorrhage
Toussia-Cohen S (2), 2022	Prospective cohort	Israel<br><br>Online questionnaire<br><br>January 2021 – November 2021	NA	*Exclusion:*<br>Chronic hypertension, chronic kidney disease, antiphospholipid syndrome, systemic lupus,	162	mRNA<br><br>Pfizer-BioNTech<br><br>Doses 1 and 2 (78)	During pregnancy (2nd, 3rd)	Fever<br>Myalgia<br>Headache<br>Fatigue

				multiple gestation, and previous preterm birth. Positive PCR SARS COV2 test before or during the study period.		Booster dose (84)		
UKHSA 27 January 2022 COVID-19 Vaccine Surveillance Report	Retrospective cohort	United Kingdom<br><br>UKHSA<br><br>January 2021 – June 2022	NA	Not reported	258639	mRNA/Viral vector<br><br>Pfizer-BioNTech<br>Moderna<br>AZD	During pregnancy (non specified)	Preterm birth <37 weeks<br>Stillbirth
Villar J, 2023	Prospective cohort	Argentina, Brazil, Egypt, France, Indonesia, Israel, Italy, Japan, Mexico, Nigeria, North Macedonia, Pakistan, Spain,	Age<br>Overweight or obesity<br>Presence or absence of any pre-existing medical condition<br>Country	*Inclusion*<br>Women with a documented diagnosis of covid-19. Live and stillborn singleton and multiple births, and newborn babies with	2886	mRNA/Viral vector/Inactivated virus<br><br>Pfizer-BioNTech<br>Moderna<br>Janssen<br>AZD<br>SinoVac	During pregnancy (non specified)	Maternal covid infection<br>Severe covid disease<br>Caesarean section<br>Hypertensive disorders<br>Preterm birth <37 weeks

		Switzerland, Türkiye, UK, Uruguay and USA<br><br>Hospitals part of the Oxford Maternal and Perinatal Health institute<br><br>November 2021 – June 2022		congenital anomalies.		Bharat Biotech<br>Sinopharm<br><br>One dose (non specified)<br>Two doses (non specified)<br>Booster dose (non specified)		
Voiniusyte A, 2022	Retrospective cohort	Lithuania<br><br>Online questionnaire<br><br>-July 2021	NA	*Inclusion*<br>Pregnant women primarily located in Lithuania who received at least one dose before giving birth. Enrollment was voluntary.	227	mRNA/Viral vector<br><br>Pfizer-BioNTech<br>Moderna<br>Janssen<br>AZD<br><br>One dose (227)<br>Two doses (157)	During pregnancy (1st, 2nd, 3rd)	Fever

Wainstock T, 2021	Retrospective cohort	Israel<br><br>Soroka University Medical Center<br><br>January 2021 – June 2021	Non specified	*Inclusion*<br>All women who delivered singletons between January and June 2021 at the Soroka University Medical Center<br><br>*Exclusion*	913	mRNA<br><br>Pfizer-BioNTech<br><br>One dose (non specified)<br>Two doses (non specified)	During pregnancy (2nd, 3rd)	Caesarean section<br>Gestational diabetes<br>Hypertensive disorders<br>Abnormal Apgar 5<br>Postpartum haemorrhage<br>Small for gestational age
Wang Y (1), 2022	Retrospective cohort	China<br><br>Peking University Third Hospital<br><br>Non specified	NA	*Inclusion:*<br>Completed gamete retrieval and embryo cryopreservation before getting vaccinated with inactivated COVID-19 vaccine.	460	Inactivated virus<br><br>Non specified<br><br>Two doses (460)	Before pregnancy	Miscarriage
Zdanowski W, 2022	Cross-sectional study	Poland	NA	*Exclusions*	169	mRNA	During pregnancy	Pain at injection site<br>Headache

		Online questionnaire<br><br>May 2021 – September 2021		Moderna and AstraZeneca vaccines.<br>First dose before pregnancy		Pfizer-BioNTech<br><br>One dose (169)<br>Two doses (121)	(non specified)	Fever<br>Myalgia<br>Fatigue
