# Supplementary material for: Effectiveness and safety of COVID-19 vaccines on maternal and perinatal outcomes: a systematic review and meta-analysis
Source: BMJ Glob Health. 2024 Apr 4;9(4):e014247. doi: 10.1136/bmjgh-2023-014247 (PMC11002410; doi:10.1136/bmjgh-2023-014247)
Supplement: Supplementary data [file bmjgh-2023-014247supp004.pdf]

Appendix 4. Description of the consensus judgments for assigning a risk of bias in each domain by the ROBINS-I tool

Table 1. Maternal SARS-CoV-2 infection

	Butt AA 2021 Cohort	Dagan N 2021	Villar J 2023	Butt AA 2021 TND	Paixao ES 2022	Schrag SJ 2022
**Bias due to confounding**						
Risk of bias judgement	Moderate	Moderate	Moderate	Low	Low	Low
Is there potential for confounding of the effect of intervention in this study?						
	Y	Y	Y	Y	Y	Y
Did the authors use an appropriate analysis method that controlled for all the importantconfounding domains?						
	Y	Y	Y	Y	Y	Y
Were confounding domains that were controlled for measured validly and reliably by the variables available in this study?						
	N	N	N	N	N	N
Did the authors control for any post-intervention variables that could have been affected by theintervention?						
	N	N	N	N	N	N
Observation:	Given that all the included articles are observational in nature, there exists a potential for confounding. While acceptable adjustment techniques were employed across all articles to mitigate this concern, the variables used for such adjustments are limited in number or lack validity and reliability. However, our assessment has led us to assign a "Low" rating to the following articles: Butt AA 2021 TND, Paixao ES 2022, and Schrag SJ 2022. This decision is based on the rationale outlined in the [WHO article], which highlights a significant aspect of the test-negative design—namely, the focus on a population with access to and utilization of medical care. This deliberate restriction serves to minimize unmeasured confounding attributed to healthcare-seeking behaviors.					
**Bias in selection of participants into the study**						
Risk of bias judgement	Low	Low	Serious	Low	Low	Low
Was the selection of participants into the study based on participant characteristics observed after the start of the intervention?						
	N	N	Y	Y	Y	Y
Do start of follow-up and start of intervention coincide for most participants?						
	PY	PY	PN	PN	PN	PN
Were adjustment techniques used that are likely to correct for the presence of selection biases?						
	NA	NA	N	N	N	N
Observation:	Through collaborative agreement, we determined that assigning a low risk of bias to this domain would be appropriate when participant selection relied on pre-intervention baseline characteristics and when the initiation of follow-up and the commencement of intervention aligned for most participants. In contrast, the study conducted by Villar J 2023 received a classification of "Serious" risk due to the lack of alignment between the start of follow-up and the initiation of intervention. Furthermore, participant selection was based on post-intervention characteristics. Regarding the TND articles, we deemed the risk of bias to be "Low." This determination stemmed from the fact that while participant selection occurs post-intervention, these designs have undergone extensive validation for assessing vaccine efficacy [WHO reference; Otra referencia].					
**Bias in classification of interventions**						
Risk of bias judgement	Low	Moderate	Low	Low	Low	Low
Were intervention groups clearly defined?						
	Y	N	Y	Y	Y	Y
Was the information used to define intervention groups recorded at the start of the intervention?						
	PY	PY	Y	PY	Y	Y
Could the classification of intervention status have been affected by knowledge of the outcome or risk of the outcome?						
	PN	PN	PN	PN	PN	PN
Observation:	We decided to score this domain as having a low risk of bias if the study described the intervention in terms of the type of vaccine used and the dose administered, which was not the case in the study by Dagan N 2021.					
**Bias due to deviations from intended interventions**						
Risk of bias judgement	Low	Low	Low	Low	Low	Low
Were there deviations from the intended intervention beyond what would be expected in usual practice?						
	N	N	N	N	N	N
Observation:	We decided to score this domain as having a low risk of bias because any deviations from the intended intervention reflected usual practice.					
**Bias due to missing data**						
Risk of bias judgement	Low	Low	Low	Low	Moderate	Moderate
Were outcome data available for all, or nearly all, participants?						
	Y	Y	Y	Y	N	N
Were participants excluded due to missing data on intervention status?						
	NI	NI	N	N	N	N
Were participants excluded due to missing data on other variables needed for the analysis?						
	NI	PY	N	N	N	N
Observation:	We've chosen to assign a "Low" rating to this section due to the adequately comprehensive nature of the data, coupled with the absence of any indications suggesting a notable divergence in the proportion or ratio of missing participant data between the intervention groups. However, for the Paixao ES 2022 and Schrag SJ 2022 items, we've opted for a "Moderate" rating. This decision arises from an insufficiency of information regarding the handling of missing data within the outcome evaluation process.					
**Bias in measurement of outcomes**						
Risk of bias judgement	Moderate	Moderate	Moderate	Low	Low	Low
Could the outcome measure have been influenced by knowledge of the intervention received?						
	Y	Y	Y	Y	Y	Y
Were outcome assessors aware of the intervention received by study participants?						
	PY	PY	PY	PY	PY	PY
Were the methods of outcome assessment comparable across intervention groups?						
	Y	Y	Y	Y	Y	Y
Were any systematic errors in the measurement of the outcome related to the intervention received?						
	N	N	N	N	N	N
Observation:	Given the observational and retrospective nature of these articles, we acknowledge that clinicians were likely aware of the patients' allocation to specific intervention groups in all instances. Moreover, this awareness might have been influenced by the patients' COVID infection status, potentially introducing bias. Consequently, a "Moderate" rating was deemed appropriate for cohort studies, considering these inherent attributes. However, for test-negative design (TND) studies, which exhibit distinct characteristics pertinent to assessing effectiveness [WHO reference], a "Low" rating was assigned due to the outlined considerations.					
**Bias in selection of the reported result**						
Risk of bias judgement	Low	Low	Low	Low	Low	Low
Is the reported effect estimate likely to be selected, on the bases of the result from... multiple outcome measurements within the outcome domain?						
	N	N	N	N	N	N
... multiple analysis of the intervention-outcome relationship?						
	N	N	N	N	N	N
... different subgroups?						
	N	N	N	N	N	N
Observation:	We decided to score this domain as having a low risk of bias because we have reported all of the results that we consider to be of interest.					
**Overall bias**						
Risk of bias judgement	Moderate	Moderate	Serious	Low	Moderate	Moderate

Table 2. Neonatal SARS-CoV-2 infection

	Carlsen EO 2022	Danino D 2022
**Bias due to confounding**		
Risk of bias judgement	Moderate	Critical
Is there potential for confounding of the effect of intervention in this study?	Y	PY
Did the authors use an appropriate analysis method that controlled for all the important confounding domains?	Y	Y
Were confounding domains that were controlled for measured validly and reliably by the variables available in this study?	N	N
Did the authors control for any post-intervention variables that could have been affected by the intervention?	N	Y
Observation:	Given that all the included articles are observational in nature, there exists a potential for confounding. While acceptable adjustment techniques were employed across all articles to mitigate this concern, the variables used for such adjustments are limited in number or lack validity and reliability. The decision to score Danino D 2022 as "High" risk is based on the use of the variable "Prematurity" as an adjustment variable, this being a post-intervention variable.	
**Bias in selection of participants into the study**		
Risk of bias judgement	Serious	Low
Was the selection of participants into the study based on participant characteristics observed after the start of the intervention?	N	Y
Do start of follow-up and start of intervention coincide for most participants?	N	PN
Were adjustment techniques used that are likely to correct for the presence of selection biases?	N	N
Observation:	Through collaborative agreement, we determined that assigning a low risk of bias to this domain would be appropriate when participant selection relied on pre-intervention baseline characteristics and when the initiation of follow-up and the commencement of intervention aligned for most participants. In contrast, the study conducted by Carlsen EO 2022 received a classification of "Serious" risk due to the lack of alignment	
**Bias in classification of interventions**		
Risk of bias judgement	Low	Low
Were intervention groups clearly defined?	Y	Y
Was the information used to define intervention groups recorded at the start of the intervention?	Y	Y
Could the classification of intervention status have been affected by knowledge of the outcome or risk of the outcome?	PN	PN
Observation:	We decided to score this domain as having a low risk of bias if the study described the intervention in terms of the type of vaccine used and the dose administered.	
**Bias due to deviations from intended interventions**		
Risk of bias judgement	Low	Low
Were there deviations from the intended intervention beyond what would be expected in usual practice?	N	N
Observation:	We decided to score this domain as having a low risk of bias because any deviations from the intended intervention reflected usual practice.	
**Bias due to missing data**		
Risk of bias judgement	Low	Low
Were outcome data available for all, or nearly all, participants?	Y	Y
Were participants excluded due to missing data on intervention status? confounding domains?	NI	NI
Were participants excluded due to missing data on other variables needed for the analysis?	N	NI
Observation:	ɔf the data, coupled with the absence of any indications suggesting a notab	
**Bias in measurement of outcomes**		
Risk of bias judgement	Low	Low
Could the outcome measure have been influenced by knowledge of the intervention received?	N	N
Were outcome assessors aware of the intervention received by study participants?	PY	PY
Were the methods of outcome assessment comparable across intervention groups?	Y	Y
Were any systematic errors in the measurement of the outcome related to the intervention received?	N	NI
Observation:	We decided by consensus	
**Bias in selection of the reported result**		
Risk of bias judgement	Low	Low
Is the reported effect estimate likely to be selected, on the bases of the result from... multiple outcome measurements within the outcome domain?	N	N
... multiple analysis of the intervention-outcome relationship?	N	N
... different subgroups?	N	N
Observation:	We decided by consensus	
**Overall bias**		
Risk of bias judgement	Moderate	Critical

Table 3. Severe covid disease

	Guedalia J 2022	Villar J 2023
**Bias due to confounding**		
Risk of bias judgement	Moderate	Moderate
Is there potential for confounding of the effect of intervention in this study?	PY	Y
Did the authors use an appropriate analysis method that controlled for all the important confounding domains?	PY	Y
Were confounding domains that were controlled for measured validly and reliably by the variables available in this study?	N	N
Did the authors control for any post-intervention variables that could have been affected by the intervention?	N	N
Observation:	We decided by consensus	
**Bias in selection of participants into the study**		
Risk of bias judgement	Low	Serious
Was the selection of participants into the study based on participant characteristics observed after the start of the intervention?	N	Y
Do start of follow-up and start of intervention coincide for most participants?	Y	PN
Were adjustment techniques used that are likely to correct for the presence of selection biases?	NA	N
Observation:	We decided by consensus	
**Bias in classification of interventions**		
Risk of bias judgement	Low	Low
Were intervention groups clearly defined?	Y	Y
Was the information used to define intervention groups recorded at the start of the intervention?	Y	Y
Could the classification of intervention status have been affected by knowledge of the outcome or risk of the outcome?	PN	PN
Observation:	We decided by consensus	
**Bias due to deviations from intended interventions**		
Risk of bias judgement	Low	Low
Were there deviations from the intended intervention beyond what would be expected in usual practice?	N	N
Observation:	We decided by consensus	
**Bias due to missing data**		
Risk of bias judgement	Low	Low
Were outcome data available for all, or nearly all, participants?	Y	Y
Were participants excluded due to missing data on intervention status? confounding domains?	NI	N
Were participants excluded due to missing data on other variables needed for the analysis?	NI	N
Observation:	We decided by consensus	
**Bias in measurement of outcomes**		
Risk of bias judgement	Low	Moderate
Could the outcome measure have been influenced by knowledge of the intervention received?	N	Y
Were outcome assessors aware of the intervention received by study participants?	PY	PY
Were the methods of outcome assessment comparable across intervention groups?	Y	Y
Were any systematic errors in the measurement of the outcome related to the intervention received?	N	N
Observation:	We decided by consensus	
**Bias in selection of the reported result**		
Risk of bias judgement	Low	Low
Is the reported effect estimate likely to be selected, on the bases of the result from... multiple outcome measurements within the outcome domain?	N	N
... multiple analysis of the intervention-outcome relationship?	N	N
... different subgroups?	N	N
Observation:	We decided by consensus	
**Overall bias**		
Risk of bias judgement	Moderate	Serious

Table 4. Maternal hospital admission

	Dagan N 2021	Guedalia J 2022	Schrag SJ 2022
**Bias due to confounding**			
Risk of bias judgement	Moderate	Moderate	Moderate
Is there potential for confounding of the effect of intervention in this study?			
	Y	PY	Y
Did the authors use an appropriate analysis method that controlled for all the important confounding domains?			
	Y	PY	Y
Were confounding domains that were controlled for measured validly and reliably by the variables available in this study?			
	N	N	N
Did the authors control for any post-intervention variables that could have been affected by theintervention?			
	N	N	N
Observation:	We decided by consensus	We decided by consensus	
**Bias in selection of participants into the study**			
Risk of bias judgement	Low	Low	Serious
Was the selection of participants into the study based on participant characteristics observed after the start of the intervention?			
	N	N	Y
Do start of follow-up and start of intervention coincide for most participants?			
	PY	Y	PN
Were adjustment techniques used that are likely to correct for the presence of selection biases?			
	NA	NA	N
Observation:	We decided by consensus	We decided by consensus	
**Bias in classification of interventions**			
Risk of bias judgement	Moderate	Low	Low
Were intervention groups clearly defined?			
	N	Y	Y
Was the information used to define intervention groups recorded at the start of the intervention? confounding domains?			
	PY	Y	Y
Could the classification of intervention status have been affected by knowledge of the outcome or risk of the outcome?			
	PN	PN	PN
Observation:	We decided by consensus	We decided by consensus	
**Bias due to deviations from intended interventions**			
Risk of bias judgement	Low	Low	Low
Were there deviations from the intended intervention beyond what would be expected in usual practice?			
	N	N	N
Observation:	We decided by consensus	We decided by consensus	
**Bias due to missing data**			
Risk of bias judgement	Low	Low	Low
Were outcome data available for all, or nearly all, participants?			
	Y	Y	N
Were participants excluded due to missing data on intervention status?			
	NI	NI	N
Were participants excluded due to missing data on other variables needed for the analysis?			
	PY	NI	N
Observation:	We decided by consensus	We decided by consensus	
**Bias in measurement of outcomes**			
Risk of bias judgement	Low	Low	Low
Could the outcome measure have been influenced by knowledge of the intervention received?			
	N	N	N
Were outcome assessors aware of the intervention received by study participants?			
	PY	PY	PY
Were the methods of outcome assessment comparable across intervention groups?			
	Y	Y	Y
Were any systematic errors in the measurement of the outcome related to the intervention received?			
	N	N	N
Observation:	We decided by consensus	We decided by consensus	
**Bias in selection of the reported result**			
Risk of bias judgement	Low	Low	Low
Is the reported effect estimate likely to be selected, on the bases of the result from... multiple outcome measurements within the outcome domain?			
	N	N	N
... multiple analysis of the intervention-outcome relationship?			
	N	N	N
... different subgroups?			
	N	N	N
Observation:	We decided by consensus	We decided by consensus	
**Overall bias**			
Risk of bias judgement	Low	Moderate	Serious

Table 5. Pregnancy-related maternal and offspring outcomes

	Fell DB (1) 2022	Goldshtein I 2022	Ibroci E 2022	Blakeway H 2021	Boelig RC 2022	Cao M 2022
**Bias due to confounding**						
Risk of bias judgement	Low	Moderate	Low	Serious	Low	Critical
Is there potential for confounding of the effect of intervention in this study?						
	Y	Y	Y	Y	Y	Y
Did the authors use an appropriate analysis method that controlled for all the importantconfounding domains?						
	Y	Y	Y	Y	Y	Y
Were confounding domains that were controlled for measured validly and reliably by the variables available in this study?						
	Y	N	Y	NI	Y	N
Did the authors control for any post-intervention variables that could have been affected by the intervention?						
	N	N	N	NI	N	PY
Observation:	Articles demonstrating a strong alignment by employing valid and dependable covariates were categorized as "Low." For those utilizing a limited subset of the crucial adjustment variables or not adhering to sound and reliable construction methods, a "Moderate" classification was assigned. In cases where the model formulation or variable selection lacked accuracy, or when relevant information was absent, an assessment of "Serious" was attributed. The most stringent rating of "Critical" was reserved for instances where post-intervention variables were employed for adjustment purposes.					
**Bias in selection of participants into the study**						
Risk of bias judgement	Low	Serious	Low	Low	Low	Serious
Was the selection of participants into the study based on participant characteristics observed after the start of the intervention?						
	N	N	N	N	N	Y
Do start of follow-up and start of intervention coincide for most participants?						
	PY	PN	PY	PY	PY	PN
Were adjustment techniques used that are likely to correct for the presence of selection biases?						
	NA	N	NA	NA	NA	NI
Observation:	By means of a collective consensus, we ascertained that attributing a domain with a low risk of bias is warranted when participant selection hinges on pre-intervention baseline characteristics, and the synchronization between the onset of follow-up and the initiation of intervention is prevalent among the majority of participants. Conversely, a study is designated as "Serious" in cases where the commencement of follow-up and the initiation of intervention do not align, and the authors have not implemented corrective techniques to mitigate this potential bias.					
**Bias in classification of interventions**						
Risk of bias judgement	Low	Moderate	Low	Low	Low	Low
Were intervention groups clearly defined?						
	Y	N	Y	Y	Y	Y
Was the information used to define intervention groups recorded at the start of the intervention?						
	Y	Y	Y	Y	Y	Y
Could the classification of intervention status have been affected by knowledge of the outcome or risk of the outcome?						
	PN	PN	PN	PN	PN	PN
Observation:	We opted to assign a "Low" risk of bias rating to this domain if the study provided a comprehensive description of the intervention, encompassing details about the specific vaccine type					
**Bias due to deviations from intended interventions**						
Risk of bias judgement	Low	Low	Low	Low	Low	Low
Were there deviations from the intended intervention beyond what would be expected in usual practice?						
	N	N	N	N	N	N
Observation:	We decided to score this domain as having a low risk of bias because any deviations from the intended intervention reflected usual practice.					
**Bias due to missing data**						
Risk of bias judgement	Low	Low	Low	Low	Low	Moderate
Were outcome data available for all, or nearly all, participants?						
	Y	Y	Y	Y	Y	PN
Were participants excluded due to missing data on intervention status?						
	N	PY	PY	Y	NI	N
Were participants excluded due to missing data on other variables needed for the analysis?						
	N	PY	PY	NI	NI	N
Observation:	We've chosen to assign a "Low" rating to this section due to the adequately comprehensive nature of the data, coupled with the absence of any indications suggesting a notable divergence in the proportion or ratio of missing participant data between the intervention groups. We decided a "Moderate" rating if there are inadequate provision of information regarding the outcome missing data.					
**Bias in measurement of outcomes**						
Risk of bias judgement	Low	Low	Low	Low	Low	Low
Could the outcome measure have been influenced by knowledge of the intervention received?						
	PN	PN	PN	PN	PN	PN
Were outcome assessors aware of the intervention received by study participants?						
	PY	PY	PY	PY	PY	PY
Were the methods of outcome assessment comparable across intervention groups?						
	Y	Y	Y	Y	Y	Y
Were any systematic errors in the measurement of the outcome related to the intervention received?						
	N	N	N	N	N	N
Observation:	We decided to score this domain as "Low" risk of bias because our safety events of interest are well defined and we can considered them as a hard outcomes unlikely to be misinterpreted					
**Bias in selection of the reported result**						
Risk of bias judgement	Low	Low	Low	Low	Low	Low
Is the reported effect estimate likely to be selected, on the bases of the result from... multiple outcome measurements within the outcome domain?						
	N	N	N	N	N	N
... multiple analysis of the intervention-outcome relationship?						
	N	N	N	N	N	N
... different subgroups?						
	N	N	N	N	N	N
Observation:	We decided to score this domain as having a low risk of bias because we have reported all of the results that we consider to be of interest.					
**Overall bias**						
Risk of bias judgement	Low	Serious	Low	Serious	Low	Critical

Table 5. Pregnancy-related maternal and offspring outcomes (continued)

	Citu IM (1) 2022	Dick A 2022	Dick A (1) 2022	Hui L (1) 2022	Magnus MC (3) 2022	Ortqvist AK 2022 Norway
**Bias due to confounding**						
Risk of bias judgement	Serious	Low	Low	Low	Critical	Moderate
Is there potential for confounding of the effect of intervention in this study?						
	Y	Y	Y	Y	Y	Y
Did the authors use an appropriate analysis method that controlled for all the importantconfounding domains?						
	PN	Y	Y	Y	Y	Y
Were confounding domains that were controlled for measured validly and reliably by the variables available in this study?						
	N	Y	Y	Y	Y	N
Did the authors control for any post-intervention variables that could have been affected by the intervention?						
	N	N	N	N	PY	N
Observation:	Articles demonstrating a strong alignment by employing valid and dependable covariates were categorized as "Low." For those utilizing a limited subset of the crucial adjustment variables or not adhering to sound and reliable construction methods, a "Moderate" classification was assigned. In cases where the model formulation or variable selection lacked accuracy, or when relevant information was absent, an assessment of "Serious" was attributed. The most stringent rating of "Critical" was reserved for instances where post-intervention variables were employed for adjustment purposes.					
**Bias in selection of participants into the study**						
Risk of bias judgement	Low	Serious	Serious	Serious	Serious	Low
Was the selection of participants into the study based on participant characteristics observed after the start of the intervention?						
	N	Y	Y	Y	Y	N
Do start of follow-up and start of intervention coincide for most participants?						
	PY	PN	PN	PN	PN	PY
Were adjustment techniques used that are likely to correct for the presence of selection biases?						
	NA	N	N	N	N	NA
Observation:	By means of a collective consensus, we ascertained that attributing a domain with a low risk of bias is warranted when participant selection hinges on pre-intervention baseline characteristics, and the synchronization between the onset of follow-up and the initiation of intervention is prevalent among the majority of participants. Conversely, a study is designated as "Serious" in cases where the commencement of follow-up and the initiation of intervention do not align, and the authors have not implemented corrective techniques to mitigate this potential bias.					
**Bias in classification of interventions**						
Risk of bias judgement	Moderate	Moderate	Low	Low	Low	Moderate
Were intervention groups clearly defined?						
	N	N	Y	Y	Y	N
Was the information used to define intervention groups recorded at the start of the intervention?						
	Y	Y	Y	Y	Y	Y
Could the classification of intervention status have been affected by knowledge of the outcome or risk of the outcome?						
	PN	PN	PN	PN	PN	PN
Observation:	We opted to assign a "Low" risk of bias rating to this domain if the study provided a comprehensive description of the intervention, encompassing details about the specific vaccine type and dosage administered. If such information was lacking, the rating was categorized as "Moderate."					
**Bias due to deviations from intended interventions**						
Risk of bias judgement	Low	Low	Low	Low	Low	Low
Were there deviations from the intended intervention beyond what would be expected in usual practice?						
	N	N	N	N	N	N
Observation:	We decided to score this domain as having a low risk of bias because any deviations from the intended intervention reflected usual					
**Bias due to missing data**						
Risk of bias judgement	Moderate	Low	Low	Low	Low	Low
Were outcome data available for all, or nearly all, participants?						
	NI	Y	Y	Y	Y	Y
Were participants excluded due to missing data on intervention status?						
	NI	Y	N	N	NI	NI
Were participants excluded due to missing data on other variables needed for the analysis?						
	NI	Y	N	N	N	N
Observation:	We've chosen to assign a "Low" rating to this section due to the adequately comprehensive nature of the data, coupled with the absence of any indications suggesting a notable divergence in the proportion or ratio of missing participant data between the intervention groups. We decided a "Moderate" rating if there are inadequate provision of information regarding the outcome missing data.					
**Bias in measurement of outcomes**						
Risk of bias judgement	Low	Low	Low	Low	Low	Low
Could the outcome measure have been influenced by knowledge of the intervention received?						
	PN	PN	PN	PN	PN	PN
Were outcome assessors aware of the intervention received by study participants?						
	PY	PY	PY	PY	PY	PY
Were the methods of outcome assessment comparable across intervention groups?						
	Y	Y	Y	Y	Y	Y
Were any systematic errors in the measurement of the outcome related to the intervention received?						
	N	N	N	N	N	N
Observation:	We decided to score this domain as "Low" risk of bias because our safety events of interest are well defined and we can considered them as a hard outcomes unlikely to be misinterpreted					
**Bias in selection of the reported result**						
Risk of bias judgement	Low	Low	Low	Low	Low	Low
likely to be selected, on the						
	N	N	N	N	N	N
... multiple analysis of the intervention-outcome relationship?						
	N	N	N	N	N	N
... different subgroups?						
	N	N	N	N	N	N
Observation:	We decided to score this domain as having a low risk of bias because we have reported all of the results that we consider to be of					
**Overall bias**						
Risk of bias judgement	Serious	Serious	Serious	Serious	Critical	Moderate

Table 5. Pregnancy-related maternal and offspring outcomes (continued)

	Ortqvist AK 2022 Sweden	Perez-Machluf R 2022	Rottenstreich M 2022	Stock S 2022	Wainstock T 2021
**Bias due to confounding**					
Risk of bias judgement	Moderate	Low	Serious	Moderate	Moderate
Is there potential for confounding of the effect of intervention in					
	Y	Y	Y	Y	Y
Did the authors use an appropriate analysis method that controlled for all the importantconfounding domains?					
	Y	Y	Y	Y	Y
Were confounding domains that were controlled for measured validly and reliably by the variables available in this study?					
	N	Y	NI	N	N
Did the authors control for any post-intervention variables that could have been affected by the intervention?					
	N	N	NI	N	N
Observation:	Articles demonstrating a strong alignment by employing valid and dependable covariates were categorized as "Low." For those utilizing a limited subset of the crucial adjustment variables or not adhering to sound and reliable construction methods, a "Moderate" classification was assigned. In cases where the model formulation or variable selection lacked accuracy, or when relevant information was absent, an assessment of "Serious" was attributed. The most stringent rating of "Critical" was reserved for instances where post-intervention variables were employed for adjustment purposes.				
**Bias in selection of participants into the study**					
Risk of bias judgement	Low	Serious	Low	Low	Serious
Was the selection of participants into the study based on participant characteristics observed after the start of the intervention?					
	N	Y	N	Y	Y
Do start of follow-up and start of intervention coincide for most participants?					
	PY	PN	PY	PY	PN
Were adjustment techniques used that are likely to correct for the presence of selection biases?					
	NA	N	NA	NA	N
Observation:	By means of a collective consensus, we ascertained that attributing a domain with a low risk of bias is warranted when participant selection hinges on pre-intervention baseline characteristics, and the synchronization between the onset of follow-up and the initiation of intervention is prevalent among the majority of participants. Conversely, a study is designated as "Serious" in cases where the commencement of follow-up and the initiation of intervention do not align, and the authors have not implemented corrective techniques to mitigate this potential bias.				
**Bias in classification of interventions**					
Risk of bias judgement	Moderate	Low	Low	Low	Moderate
Were intervention groups clearly defined?					
	N	Y	Y	Y	N
Was the information used to define intervention groups recorded at the start of the intervention?					
	Y	Y	Y	Y	Y
Could the classification of intervention status have been affected by knowledge of the outcome or risk of the outcome?					
	PN	PN	PN	PN	PN
Observation:	We opted to assign a "Low" risk of bias rating to this domain if the study provided a comprehensive description of the intervention, encompassing details about the specific vaccine type and dosage administered. If such information was lacking, the rating was categorized as "Moderate."				
**Bias due to deviations from intended interventions**					
Risk of bias judgement	Low	Low	Low	Low	Low
Were there deviations from the intended intervention beyond what would be expected in usual practice?					
	N	N	N	N	N
Observation:	We decided to score this				
**Bias due to missing data**					
Risk of bias judgement	Low	Low	Moderate	Low	Low
Were outcome data available for all, or nearly all, participants?					
	Y	Y	NI	Y	Y
Were participants excluded due to missing data on intervention status?					
	NI	Y	NI	NI	Y
Were participants excluded due to missing data on other variables needed for the analysis?					
	N	N	NI	N	NI
Observation:	We've chosen to assign a "Low" rating to this section due to the adequately comprehensive nature of the data, coupled with the absence of any indications suggesting a notable divergence in the proportion or ratio of missing participant data between the intervention groups. We decided a "Moderate" rating if there are inadequate provision of information regarding the outcome missing data.				
**Bias in measurement of outcomes**					
Risk of bias judgement	Low	Low	Low	Low	Low
Could the outcome measure have been influenced by knowledge of the intervention received?					
	PN	PN	PN	PN	PN
Were outcome assessors aware of the intervention received by study participants?					
	PY	PY	PY	PY	PY
Were the methods of outcome assessment comparable across intervention groups?					
	Y	Y	Y	Y	Y
Were any systematic errors in the measurement of the outcome related to the intervention received?					
	N	N	N	N	N
Observation:	We decided to score this domain as "Low" risk of bias because our safety events of interest are well defined and we can considered them as a hard outcomes unlikely to be misinterpreted				
**Bias in selection of the reported result**					
Risk of bias judgement	Low	Low	Low	Low	Low
Is the reported effect estimate likely to be selected, on the bases					
	N	N	N	N	N
... multiple analysis of the intervention-outcome relationship?					
	N	N	N	N	N
... different subgroups?					
	N	N	N	N	N
Observation:	We decided to score this domain as having a low risk of bias because we have reported all of the results that we consider to be of interest.				
**Overall bias**					
Risk of bias judgement	Moderate	Serious	Serious	Moderate	Serious
