# Supplementary material for: Effectiveness and safety of COVID-19 vaccines on maternal and perinatal outcomes: a systematic review and meta-analysis
Source: BMJ Glob Health. 2024 Apr 4;9(4):e014247. doi: 10.1136/bmjgh-2023-014247 (PMC11002410; doi:10.1136/bmjgh-2023-014247)

Appendix 5. Vaccine effectiveness for SARS-CoV-2 infection-related outcomes (unadjusted analysis)

Outcome and Author	No of events/No of vaccinated women	No of events/No of unvaccinated women	Odds Ratio (95% CI)
**Maternal SARS-CoV-2 infection**			
Blakeway H 2021	2/140	16/1188	1.06 (0.24, 4.67)
Bleicher I 2021	3/202	8/124	0.22 (0.06, 0.84)
Guedalia J 2022	2629/51942	2090/30627	0.73 (0.69, 0.77)
Guedalia J 2022	1747/8612	1089/8282	1.68 (1.55, 1.83)
Magnus MC (3) 2022	1513/28506	10988/129015	0.60 (0.57, 0.64)
Piekos SN 2022	916/26792	3394/48492	0.47 (0.44, 0.51)
Theiler RN 2021	0/140	210/1862	0.03 (0.00, 0.45)
Dagan N 2021	131/10861	235/10861	0.55 (0.45, 0.69)
Carlsen EO 2022	363/9739	1036/11904	0.41 (0.36, 0.46)
Goldshtein I 2021	118/7530	202/7530	0.58 (0.46, 0.73)
Villar J 2023	525/1598	632/1732	0.85 (0.74, 0.98)
Subtotal (I-squared = 98.5%, p = 0.000)	7947/146062	19900/251617	0.63 (0.47, 0.85)
**Offspring SARS-CoV-2 infection**			
Carlsen EO 2022	25/4696	146/9759	0.76 (0.65, 0.88)
Carlsen EO 2022	385/9616	350/6728	0.35 (0.23, 0.54)
Halasa NB (1) 2022	87/234	450/815	0.48 (0.36, 0.65)
Subtotal (I-squared = 87.6%, p = 0.000)	497/14546	946/17302	0.52 (0.33, 0.82)
**Severe covid disease**			
Guedalia J 2022	3/51942	64/30627	0.03 (0.01, 0.09)
Guedalia J 2022	1/8612	5/8282	0.19 (0.02, 1.65)
COVID-NET nov 2021	1/11	44/322	0.63 (0.08, 5.06)
Blakeway H 2021	8/131	11/393	2.26 (0.89, 5.74)
Butt AA 2021	0/103	9/1117	0.56 (0.03, 9.75)
Dagan N 2021	0/10861	1/10861	0.33 (0.01, 8.18)
Kim H 2022	0/39	4/185	0.51 (0.03, 9.68)
Paganoti CDF (2) 2022	44/187	740/1979	0.51 (0.36, 0.73)
Theiler RN 2021	1/140	2/1862	6.69 (0.60, 74.24)
Villar J 2023	36/1598	85/1732	0.45 (0.30, 0.66)
Rottenstreich M 2022	0/712	0/1063	Insufficient data
Subtotal (I-squared = 80.9%, p = 0.000)	94/74336	965/58423	0.47 (0.22, 0.97)
**Maternal hospital admission**			
Goldstein I 2021	13/7530	23/7530	0.56 (0.29, 1.11)
Dagan N 2021	1/10861	7/10861	0.14 (0.02, 1.16)
Subtotal (I-squared = 92.0%, p = 0.000)	14/18391	30/18391	0.41 (0.13, 1.28)
**Maternal death**			
Guedalia J 2022	0/51942	1/30627	0.20 (0.01, 4.82)
Paganoti CDF (2) 2022	6/200	294/2084	0.21 (0.10, 0.47)
Piekos SN 2022	3/1178	3/3394	2.88 (0.58, 14.26)
Villar J 2023	1/1973	1/1100	0.56 (0.03, 8.90)
COVID-NET nov 2021	0/11	0/322	Insufficient data
Dagan N 2021	0/10861	0/10861	Insufficient data
Goldshtein I 2021	0/7530	0/7530	Insufficient data
Guedalia J 2022	0/8612	0/8282	Insufficient data
Theiler RN 2021	0/138	0/1652	Insufficient data
Subtotal (I-squared = 64.4%, p = 0.038)	10/82445	299/65852	0.53 (0.12, 2.47)

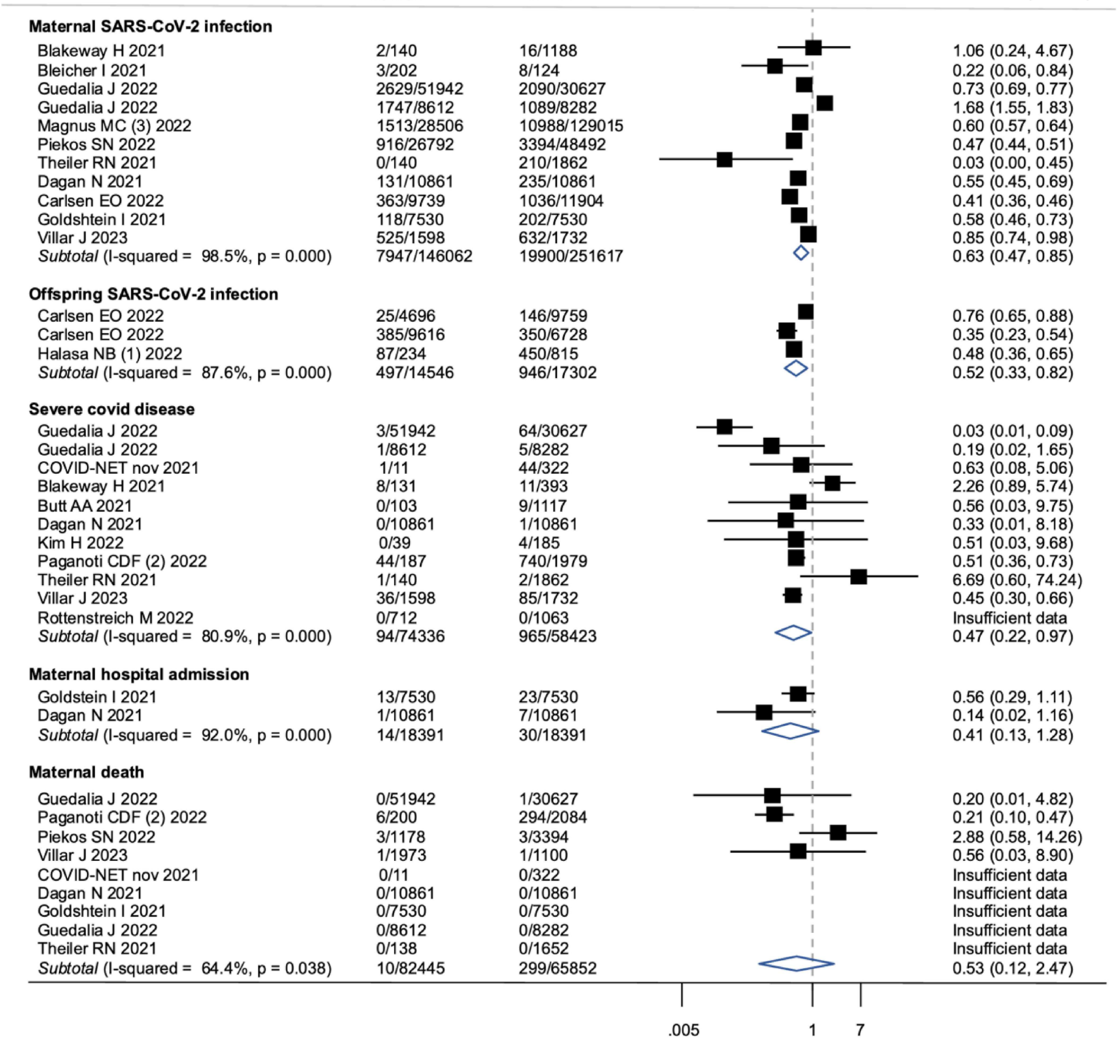

Supplement: Supplementary data [file bmjgh-2023-014247supp005.pdf]
