# Supplementary material for: Effectiveness and safety of COVID-19 vaccines on maternal and perinatal outcomes: a systematic review and meta-analysis
Source: BMJ Glob Health. 2024 Apr 4;9(4):e014247. doi: 10.1136/bmjgh-2023-014247 (PMC11002410; doi:10.1136/bmjgh-2023-014247)

# Appendix 6. Effect of vaccine for pregnancy-related maternal outcomes (adjusted and unadjusted analysis)

| Outcomes | No of studies | No of events / No of vaccinated women | No of events / No of unvaccinated women | Odds Ratio (95% CI) | $I^2$ |
|---|---|---|---|---|---|
| **Miscarriage** | | | | | |
| Adjusted | 4 | > 1874/20719 | > 2233/22746 | 0.96 (0.90, 1.04) | 0.0% |
| Unadjusted | 3 | 21/389 | 46/724 | 1.60 (0.70, 1.91) | 0.0% |
| **Preterm birth < 37 weeks** | | | | | |
| Adjusted | 5 | 865/15570 | 733/9946 | 0.79 (0.59, 1.06) | 68.3% |
| Unadjusted | 21 | 21179/373909 | 42281/730134 | 0.90 (0.83, 0.97) | 75.0% |
| **Caesarean section** | | | | | |
| Adjusted | 6 | 3339/16565 | 2492/13627 | 0.91 (0.85, 0.98) | 0.0% |
| Unadjusted | 15 | 17395/56221 | 39662/131923 | 1.11 (1.03, 1.20) | 48.6% |
| **Postpartum haemorrhage** | | | | | |
| Adjusted | 5 | 936/13325 | 447/13167 | 1.49 (0.91, 2.44) | 86.7% |
| Unadjusted | 6 | 865/25496 | 2569/79197 | 0.82 (0.68, 1.00) | 0.0% |
| **Gestational diabetes** | | | | | |
| Adjusted | 1 | 222/2305 | 275/3313 | 1.10 (0.90, 1.30) | Not applicable |
| Unadjusted | 11 | 10687/132636 | 10371/130683 | 1.04 (0.89, 1.21) | 94.2% |
| **Hypertensive disorders** | | | | | |
| Adjusted | 5 | > 150/7219 | > 257/8520 | 1.11 (0.87, 1.43) | 0.0% |
| Unadjusted | 10 | 3704/104083 | 3862/113403 | 1.13 (1.02, 1.25) | 49.0% |

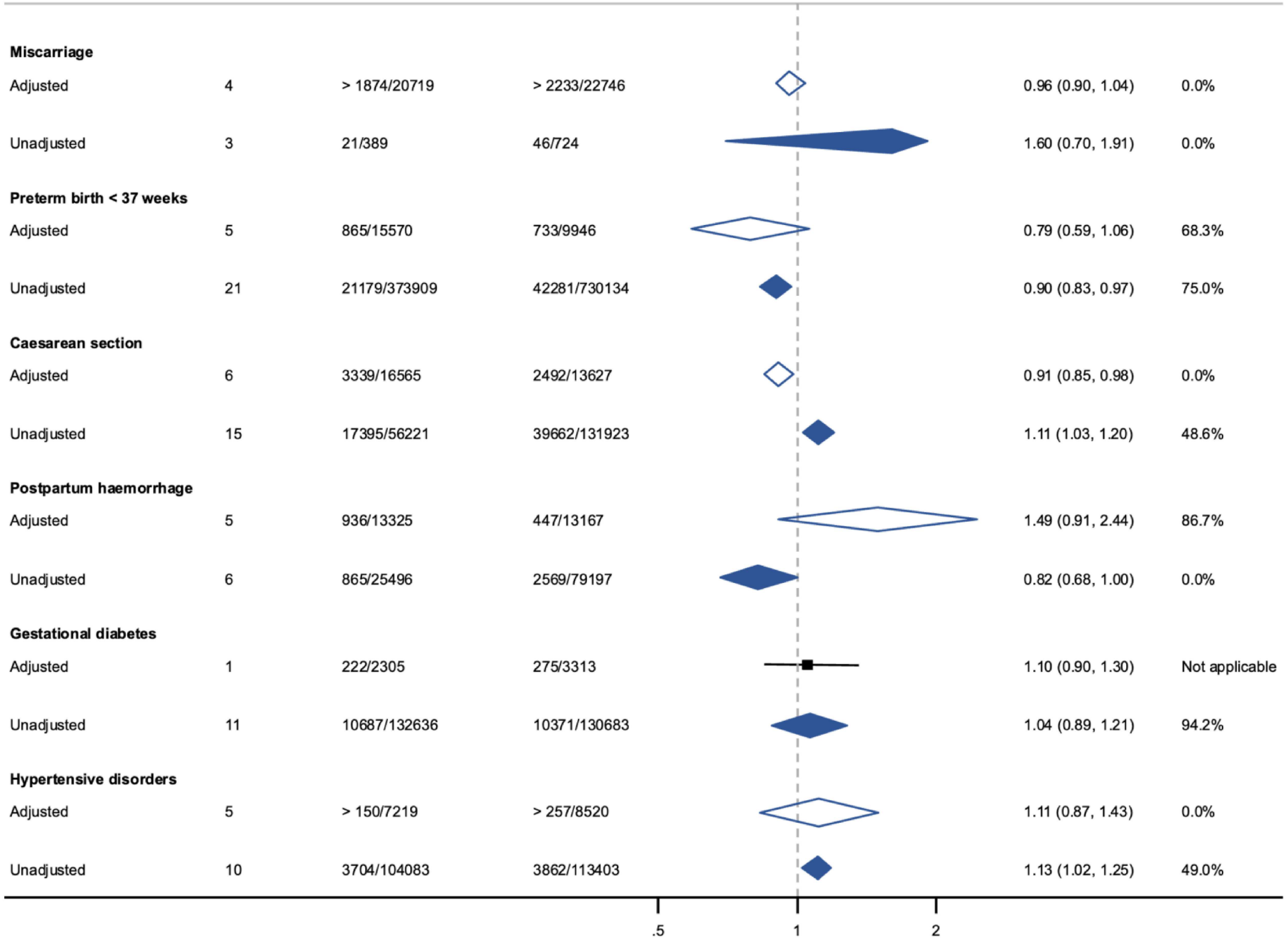

Supplement: Supplementary data [file bmjgh-2023-014247supp006.pdf]
