# Supplementary material for: Effectiveness and safety of COVID-19 vaccines on maternal and perinatal outcomes: a systematic review and meta-analysis
Source: BMJ Glob Health. 2024 Apr 4;9(4):e014247. doi: 10.1136/bmjgh-2023-014247 (PMC11002410; doi:10.1136/bmjgh-2023-014247)

Appendix 7. Effect of vaccine for pregnancy-related offspring outcomes (adjusted and unadjusted analysis)

Outcomes	No of studies	No of events / No of vaccinated women	No of events / No of unvaccinated women	Odds Ratio (95% CI)	I^2
**Stillbirth**					
Adjusted	2	35/11987	54/5920	0.38 (0.09, 1.59)	89.4%
Unadjusted	11	1120/344258	2474/680694	0.78 (0.65, 0.92)	36.5%
**Admission to neonatal intensive care unit**					
Adjusted	4	2751/41545	11157/132433	0.88 (0.71, 1.08)	37.9%
Unadjusted	9	2625/26760	10208/81774	0.82 (0.79, 0.86)	0.0%
**Low 5-minutes Apgar score < 7**					
Adjusted	4	643/40773	2460/138261	0.89 (0.73, 1.08)	29.3%
Unadjusted	9	487/30324	1616/83216	0.89 (0.81, 0.99)	0.0%
**Small for gestational age**					
Adjusted	6	2764/34304	11514/138179	0.96 (0.90, 1.02)	0.0%
Unadjusted	8	5751/69977	7150/83836	0.99 (0.95, 1.03)	0.0%

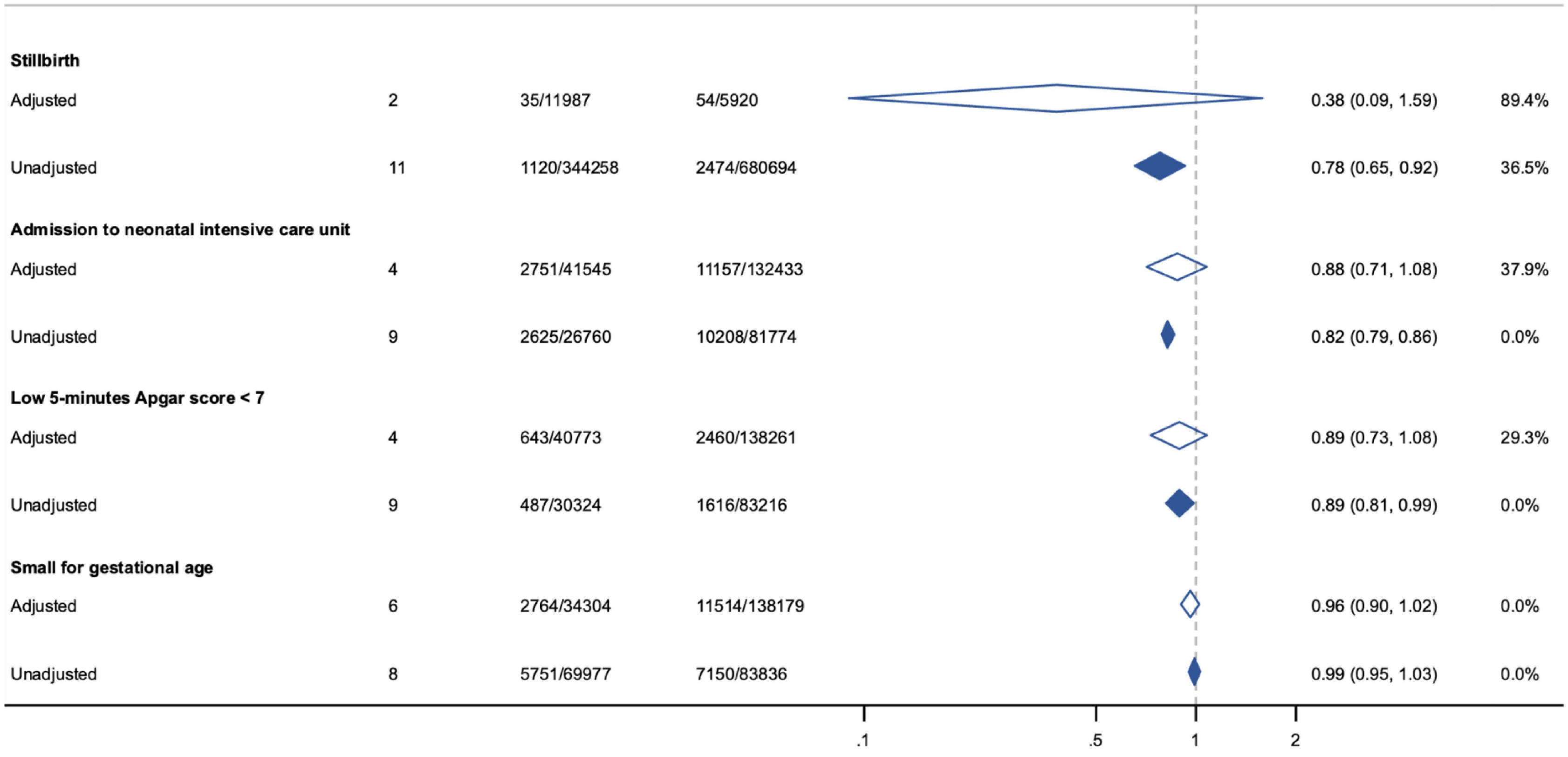

Supplement: Supplementary data [file bmjgh-2023-014247supp007.pdf]
