# Supplementary material for: Effectiveness and safety of COVID-19 vaccines on maternal and perinatal outcomes: a systematic review and meta-analysis
Source: BMJ Glob Health. 2024 Apr 4;9(4):e014247. doi: 10.1136/bmjgh-2023-014247 (PMC11002410; doi:10.1136/bmjgh-2023-014247)

Appendix 8. Effect of vaccines on pregnancy-related maternal outcomes (adjusted individual studies)

Outcome and Author	No of events/ No of vaccinated women	No of events/No of unvaccinated women	Maternal age	Gestational age	BMI	Education	Diabetes	Chronic hypertension	Meassure	Estimate (95% CI)
**Miscarriage**										
Cao M 2022	34/300	84/736	■						OR	0.99 (0.65, 1.52)
Rottenstreich M 2022	Not reported/712	Not reported/1063							OR	1.05 (0.78, 1.40)
Stock S 2022	1716/18780	1878/18780	■	■					OR	0.96 (0.88, 1.04)
Citu IM (1) 2022	124/927	271/2167	■		■		■	■	OR	0.94 (0.66, 1.05)
Subtotal (I-squared = 0.0%, p = 0.941)	> 329/20719	> 2233/22746							OR	0.96 (0.90, 1.04)
**Preterm birth < 37 weeks**										
Boelig RC 2022	5/49	13/198	■		■		■	■	OR	1.75 (0.46, 6.67)
Dick A (1) 2022	14/294	233/3368	■		■		■		OR	0.67 (0.37, 1.23)
Dick A 2022	127/2305	204/3313	■		■				OR	0.93 (0.76, 1.21)
Hui L (1) 2022	495/9682	239/2607	■		■		■		OR	0.60 (0.51, 0.71)
Perez-Machluf R 2022	224/3240	44/460	■	■	■		■	■	OR	0.94 (0.62, 1.44)
Subtotal (I-squared = 68.3%, p = 0.013)	865/15570	733/9946							OR	0.79 (0.59, 1.06)
Goldshtein I 2022	699/16738	358/7452	■						RR	0.95 (0.83, 1.10)
**Caesarean section**										
Dick A (1) 2022	53/294	558/3368	■		■		■		OR	1.04 (0.75, 1.46)
Dick A 2022	358/2305	529/3313	■		■				OR	0.94 (0.75, 1.46)
Hui L (1) 2022	1829/9682	529/2607	■		■		■		OR	0.90 (0.81, 0.99)
Perez-Machluf R 2021	877/3240	141/460	■	■	■		■	■	OR	0.86 (0.64, 1.14)
Wainstock T 2021	182/913	601/3486	■						OR	0.93 (0.75, 1.16)
Blakeway H 2021	40/131	134/393							OR	0.85 (0.55, 1.30)
Subtotal (I-squared = 0.0%, p = 0.956)	3339/16565	2492/13627							OR	0.91 (0.85, 0.98)
Fell DB (1) 2022	6988/22660	8583/30115	■		■		■	■	RR	0.97 (0.94, 1.00)
Ibroci E 2022	99/250	541/1544	■		■		■	■	RR	0.80 (0.60, 1.10)
Subtotal (I-squared = 34.9%, p = 0.215)	7087/22910	9124/31659							RR	0.94 (0.81, 1.08)
**Postpartum haemorrhage**										
Dick A (1) 2022	28/294	108/3368	■		■		■		OR	3.88 (2.41, 6.25)
Dick A 2022	79/2305	104/3313	■		■				OR	1.20 (0.85, 1.66)
Hui L (1) 2022	806/9682	205/2607	■		■		■		OR	0.97 (0.83, 1.13)
Wainstock T 2021	10/913	30/3486	■						OR	1.46 (0.63, 3.38)
Blakeway H 2021	13/131	33/393							OR	1.20 (0.61, 2.35)
Subtotal (I-squared = 86.7%, p = 0.000)	936/13325	447/13167							OR	1.49 (0.91, 2.44)
Fell DB (1) 2022	677/22660	1008/30115	■		■		■	■	RR	0.90 (0.81, 1.00)
**Gestational diabetes**										
Dick A 2022	222/2305	275/3313	■		■				OR	1.10 (0.90, 1.30)
Ortqvist AK 2022 Norway	2299/43591	2113/33047	■						RR	1.18 (1.13, 1.22)
Ortqvist AK 2022 Sweden	1195/20931	1275/17516	■						RR	1.16 (1.12, 1.20)
Subtotal (I-squared = 0.0%, p = 0.516)	3494/64522	3388/50563							RR	1.17 (1.14, 1.20)
**Hypertensive disorders**										
Boelig RC 2022	10/49	42/198	■		■		■	■	OR	1.05 (0.45, 2.41)
Dick A 2022	25/2305	44/3313	■		■				OR	0.82 (0.48, 1.38)
Perez-Machluf R 2022	65/3240	6/460	■	■	■		■	■	OR	1.30 (0.70, 2.42)
Rottenstreich M 2022	Not reported/712	Not reported/1063							OR	2.13 (0.79, 5.73)
Wainstock T 2021	50/913	165/3486	■						OR	1.13 (0.78, 1.62)
Subtotal (I-squared = 0.0%, p = 0.525)	> 150/7219	> 257/8520							OR	1.11 (0.87, 1.43)
Ortqvist AK 2022 Norway	1662/43591	1005/33047	■						RR	0.91 (0.85, 0.97)
Ortqvist AK 2022 Sweden	669/20931	480/17516	■						RR	0.87 (0.83, 0.91)
Subtotal (I-squared = 0.0%, p = 0.516)	2331/64522	1485/50563							RR	0.88 (0.85, 0.92)

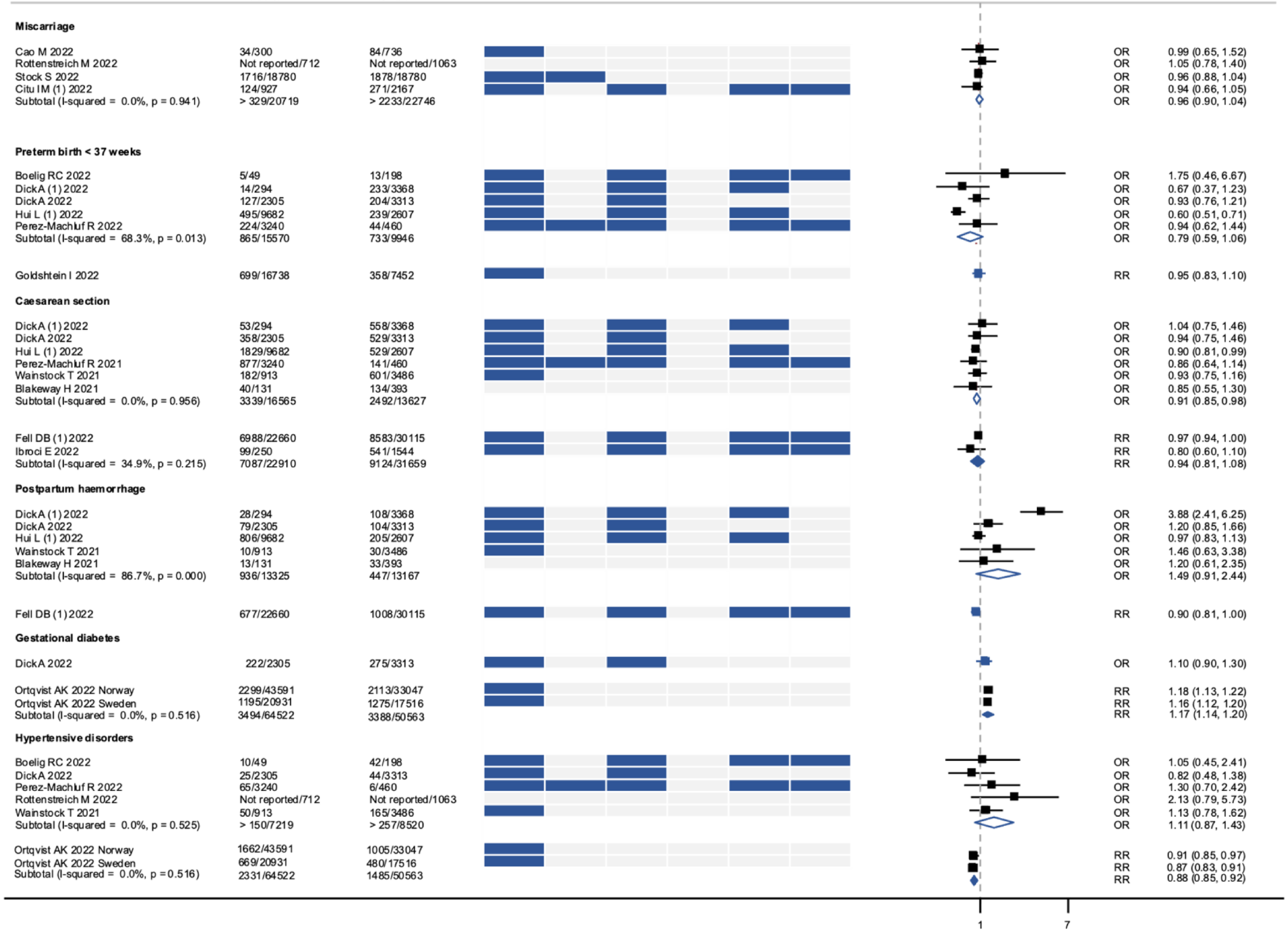

Supplement: Supplementary data [file bmjgh-2023-014247supp008.pdf]
