# Supplementary material for: Effectiveness and safety of COVID-19 vaccines on maternal and perinatal outcomes: a systematic review and meta-analysis
Source: BMJ Glob Health. 2024 Apr 4;9(4):e014247. doi: 10.1136/bmjgh-2023-014247 (PMC11002410; doi:10.1136/bmjgh-2023-014247)

Appendix 9. Vaccine safety for pregnancy-related offspring outcomes (adjusted individual studies)

Outcome and Author	No of events/No of vaccinated women	No of events/No of unvacinated women	Maternal age	Gestational age	BMI	Education	Diabetes	Chronic hypertension	Meassure	Estimate (95% CI)
**Stillbirth**										
Dick A 2022	20/2305	33/3313	■		■				OR	0.78 (0.42, 1.44)
Hui L (1) 2022	15/9682	21/2607	■		■		■		OR	0.18 (0.09, 0.37)
Subtotal (I-squared = 89.4%, p = 0.002)	35/11987	54/5920							OR	0.38 (0.09, 1.59)
**Admission to neonatal intensive care unit**										
Hui L (1) 2022	236/9682	106/2607	■		■		■		OR	0.70 (0.53, 0.91)
Magnus MC (3) 2022	2419/28492	11011/128973	■	■		■	■	■	OR	0.97 (0.86, 1.10)
Perez-Machluf R 2022	89/3240	16/460	■	■	■		■	■	OR	1.06 (0.54, 2.10)
Blakeway H 2021	7/131	24/393							OR	0.86 (0.36, 2.06)
Subtotal (I-squared = 37.9%, p = 0.185)	2751/41545	11157/132433							OR	0.88 (0.71, 1.08)
Fell DB (1) 2022	2508/22660	3852/30115	■		■		■	■	RR	0.92 (0.87, 0.97)
Ibroci E 2022	25/250	152/1544	■		■		■	■	RR	0.90 (0.50, 1.70)
Subtotal (I-squared = 0.0%, p = 0.944)	2533/22910	4004/31659							RR	0.92 (0.87, 0.97)
**Low 5-minutes Apgar score <7**										
Dick A (1) 2022	2/294	257/3368	■		■		■		OR	0.27 (0.04, 1.98)
Dick A 2022	42/2305	63/3313	■		■				OR	0.92 (0.60, 1.43)
Hui L (1) 2022	170/9682	80/2607	■		■		■		OR	0.72 (0.51, 1.01)
Magnus MC (3) 2022	429/28492	2060/128973	■	■		■	■	■	OR	0.97 (0.87, 1.08)
Subtotal (I-squared = 29.3%, p = 0.236)	643/40773	2460/138261							OR	0.89 (0.73, 1.08)
Fell DB (1) 2022	403/22334	508/29588	■		■		■	■	RR	0.88 (0.77, 1.01)
**Small for gestational age**										
Dick A (1) 2022	20/294	235/3368	■		■		■		OR	1.10 (0.68, 1.82)
Dick A 2022	142/2305	233/3313	■		■				OR	0.81 (0.65, 1.05)
Magnus MC (3) 2022	2143/27421	10821/127159	■	■		■	■	■	OR	0.97 (0.90, 1.04)
Perez-Machluf R 2022	417/3240	49/460	■	■	■		■	■	OR	1.01 (0.66, 1.55)
Wainstock T 2021	26/913	131/3486	■						OR	0.79 (0.48, 1.31)
Blakeway H 2021	16/131	45/393							OR	1.07 (0.58, 1.97)
Subtotal (I-squared = 0.0%, p = 0.692)	2764/34304	11514/138179							OR	0.96 (0.90, 1.02)
Goldshtein I 2022	1053/16738	473/7452	■						RR	0.97 (0.87, 1.08)
**Neonatal death**										
Goldshtein I 2022	24/16738	13/7452	■						RR	0.84 (0.43, 1.72)

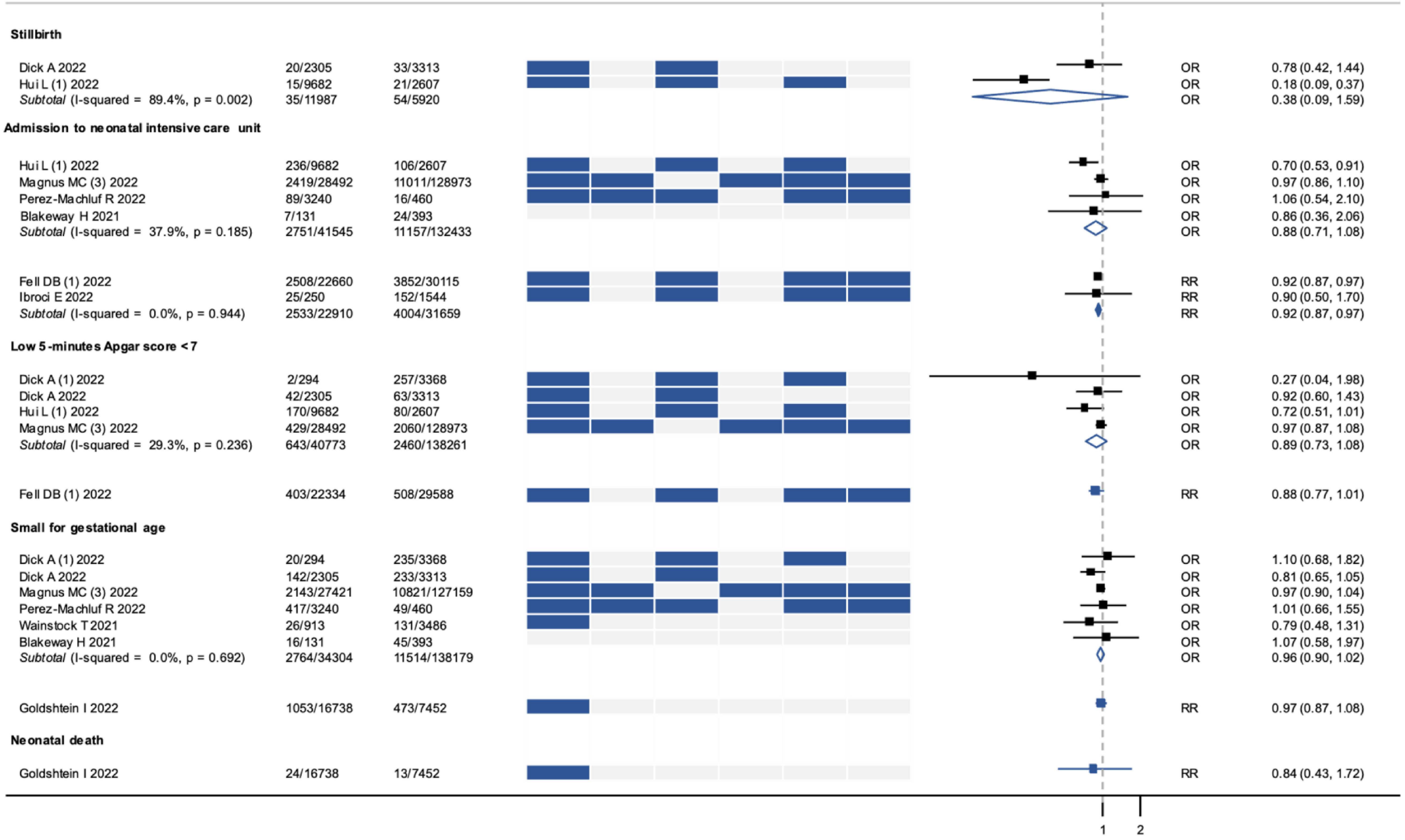

Supplement: Supplementary data [file bmjgh-2023-014247supp009.pdf]
